# Scalable synthesis and structural characterization of reversible KLK6 inhibitors†

**DOI:** 10.1039/d2ra04670a

**Published:** 2022-09-21

**Authors:** Andreas Baumann, Daniel Isak, Jasmin Lohbeck, Pravin Kumar Ankush Jagtap, Janosch Hennig, Aubry K. Miller

**Affiliations:** Cancer Drug Development Group, German Cancer Research Center (DKFZ) Im Neuenheimer Feld 280 69120 Heidelberg Germany aubry.miller@dkfz.de; Structural and Computational Biology Unit, European Molecular Biology Laboratory (EMBL) 69117 Heidelberg Germany; Chair of Biochemistry IV, Biophysical Chemistry, University of Bayreuth 95447 Bayreuth Germany; German Cancer Consortium (DKTK) Im Neuenheimer Feld 280 69120 Heidelberg Germany

## Abstract

Scalable asymmetric syntheses of two kallikrein-related protease 6 (KLK6) inhibitors are reported. The inhibitors are assembled by linking enantiomerically enriched fragments *via* amide bond formation, followed by conversion of a cyano group to an amidine. One fragment, an amine, was prepared using the Ellman auxiliary, and a lack of clarity in the literature regarding the stereochemical outcome of this reaction was solved *via* X-ray crystallographic analysis of two derivatives. Complexes of the inhibitors bound to human KLK6 were solved by X-ray crystallography, revealing the binding poses.

## Abstract 2

We report scalable syntheses of two potent and selective kallikrein related peptidase 6 (KLK6) inhibitors, as well as X-ray crystal structures of both inhibitors as protein-ligand complexes.

## Graphical abstract

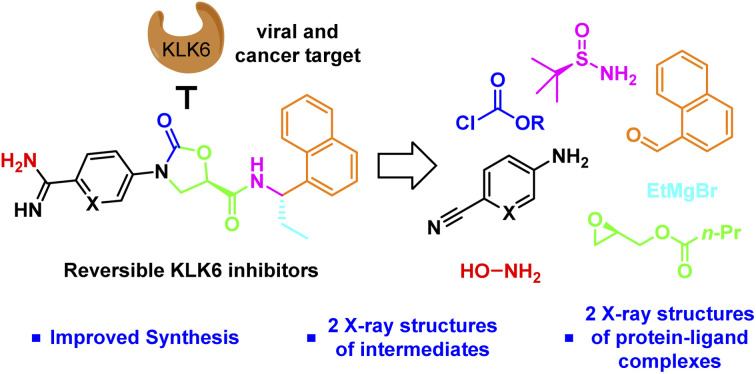

Proteases are classical drug targets for the treatment of viral infections, and the development of protease inhibitors has dramatically transformed the way in which patients infected with viruses like hepatitis C and HIV can be treated.^1^ Very recently, the protease inhibitor nirmatrelvir was developed to treat patients infected with the SARS-Cov-2 virus, giving hope that recovery from infection will become more manageable.^2^ In the above mentioned cases, the protease targets are encoded by the virus itself and are required for replication, making them attractive targets because selective inhibition should not affect any human protease targets. There is growing evidence, however, that viruses also take advantage of host proteases to assist in critical steps of the viral cycle, such as viral entry into the host cell as well as viral replication.^3^

The kallikrein-related proteases are a family of secreted serine proteases, a number of which have been implicated in viral replication.^4^ KLK6, associated with neurological diseases like MS, Alzheimer's,^5^ and Parkinson's,^6^ and with the migration and invasion, and thereby metastasis, of tumor cells,^7,8^ has also recently been linked to the zoster (chickenpox) virus.^9,10^ Infected keratinocytes were found to overexpress KLK6 and MDM2, and their overexpression was directly linked to viral propagation. The use of validated MDM2 chemical probes demonstrated that MDM2 inhibition reduces viral propagation. This was not done with KLK6 inhibitors, potentially because small molecule tools/probes for KLK6 are not yet widely available.^11^

We recently reported the synthesis and biological characterization of highly potent benzamidine KLK6 inhibitors DKFZ-917 (1) and DKFZ-918 (2), with pIC_50_ values of 8.6 and 8.3, respectively, and good selectivity over related serine proteases (Fig. 1).^12^ In order to perform more advanced preclinical experiments with these substances, and to be able to make them freely available to other researchers, we required larger quantities than were originally prepared in the hit-to-lead optimization process. We were cognizant that the original route was not optimized and decided to revisit the synthesis with the goal to establish a robust, scalable source of 1 and 2, with a reduced number of chromatographic separations. This required efficient asymmetric syntheses of acids 3/4 and amine 5, as well as the late-stage conversion of a cyano group to a benzamidine.

**Fig. 1:**
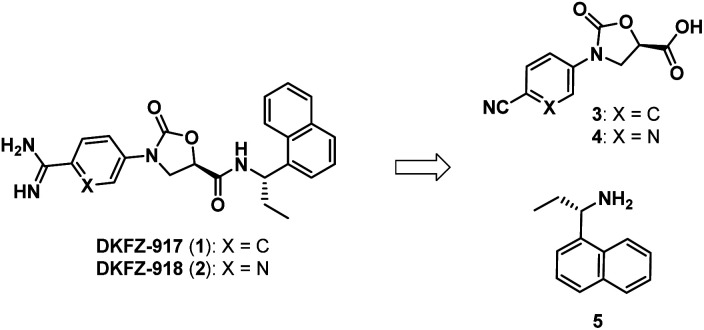
Benzamidine-containing KLK6 inhibitors.

Our synthesis of acid 3 begins with conversion of aniline 6 to methyl carbamate 7, as opposed to the corresponding benzyl carbamate, which we had used in our previous report (Scheme 1). This has the advantage of being more atom economical, while also producing methyl butyrate as a volatile byproduct in the next step as opposed to benzyl butyrate. The second step begins with stoichiometric deprotonation of the carbamate NH with a strong base. When using *n*-BuLi on larger scales, we sometimes observed addition of the alkyl lithium to the cyano group, giving a butyl ketone. Switching to the non-nucleophilic base LiHDMS, however, reliably afforded oxazolidinone 8 after crystallization from a mixture of EtOAc and *n*-hexane. Oxidation of the primary hydroxyl group in 8 with TEMPO/PhI(OAc)_2_ to give acid 3 proceeded slowly but cleanly, requiring no chromatographic purification.^13^ The synthesis of acid 4 proceeded analogously, except for the fact that benzyl carbamate 10 was required in the oxazolidinone-forming reaction. To our surprise, when using the corresponding methyl carbamate, we only recovered starting material at the end of the reaction. Regardless, oxazolidinone 11 could be purified *via* crystallization and advanced to acid 4 uneventfully.

**Scheme 1:**
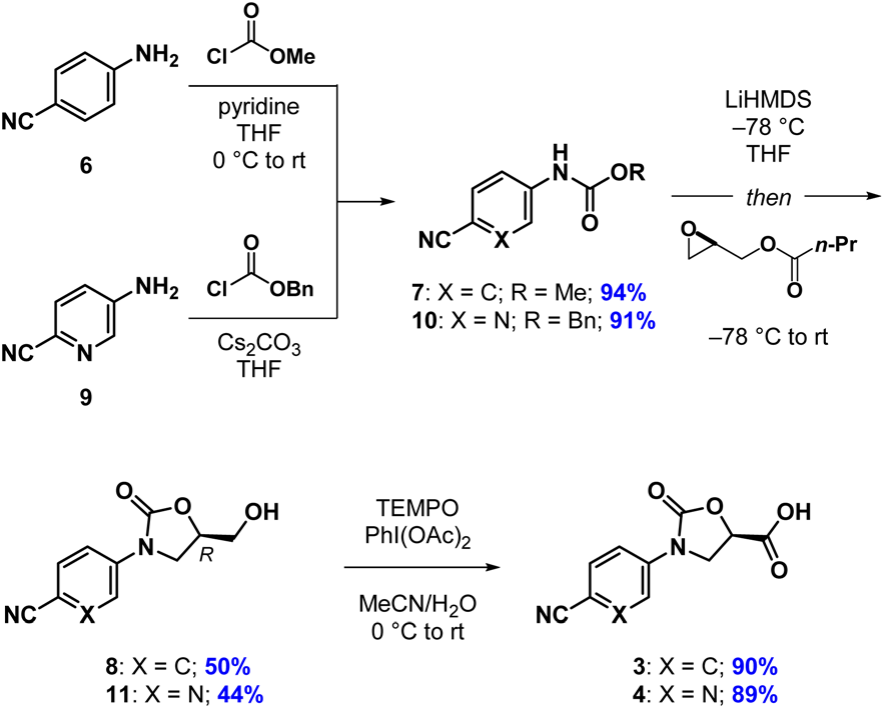
Synthesis of acids 3 and 4.

The next task involved a reliable synthesis of amine 5 with high enantiopurity. We had used Ellman's *tert*-butanesulfinamide auxiliary^14^ to synthesize many chiral amines in our initial medicinal chemistry campaign. Some reactions, including the one for 5, gave mixtures of sulfinamide diastereomers that required separation; we assigned their stereochemical configuration only *via* spectroscopic correlation to related compounds in the series and on the basis of activity in biochemical assays. For this reason, as well as the fact that we found discrepancies between our own data with other reports toward 5 (*vide infra*), we re-examined this reaction sequence in detail.

We began by adding a solution of EtMgBr in Et_2_O to (*S*)-12 in toluene at −78 °C, followed by slowly warming the reaction mixture to rt (Table 1, entry 1). After workup, NMR analysis of the crude reaction mixture revealed a 57 : 33 : 10 ratio of 13, 14, and the corresponding reduced imine 15, respectively (Table 1). We next attempted the reaction under “standard” Ellman conditions (2.5 equiv. of EtMgBr in Et_2_O added to a solution of 12 in CH_2_Cl_2_ at −48 °C, then warming to rt) and found the reaction to be extremely sluggish, taking ∼24 h to go to completion. In contrast to the first reaction, under these conditions the diastereomer predicted by the Ellman model for non-coordinating solvents was the major product, albeit with a relatively poor ratio of 24 : 59 : 17 of 13, 14, and 15, respectively. We therefore conducted the reaction in THF, a solvent generally associated with lower diastereoselectivity with the Ellman auxiliary. We were pleased that 12 was consumed within 10 min and the reaction was highly selective, providing a 90 : 10 ratio of 13 and 14, respectively, with no 15 formed.

**Table 1:** Screening of reaction conditions

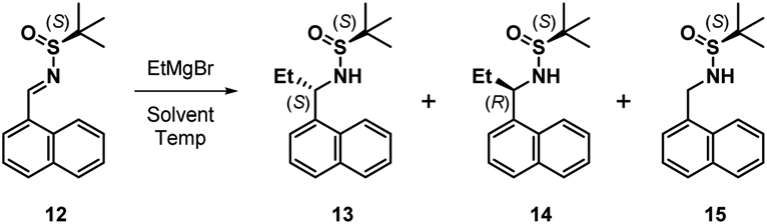				
Entry	Solvent	Temp	Time	Product ratio (13 : 14 : 15)
1	Toluene	−78 °C to rt	6 h	57 : 33 : 10
2	CH_2_Cl_2_	−48 °C to rt	24 h	24 : 59 : 17
3	THF	−78 °C	10 min	90 : 10 : 0

We observed that diagnostic signals in the ^1^H NMR spectra of 13 and 14 are consistent with literature reports, wherein the compounds bearing a methyl, as opposed to ethyl, groups were prepared using complementary approaches.^15–17^ In contrast, compound 13 had an identical ^1^H NMR spectrum to what was recently reported for 14, causing some concern about our assignment.^18^ In order to unambiguously assign the relative configuration of 13, we solved its structure *via* X-ray crystallography (Fig. 2).^19^ This confirmed the (*S*,*S*) configuration of 13, highlighting the subtlety of Ellman chemistry with respect to substrate structure and solvent.^20^

**Fig. 2:**
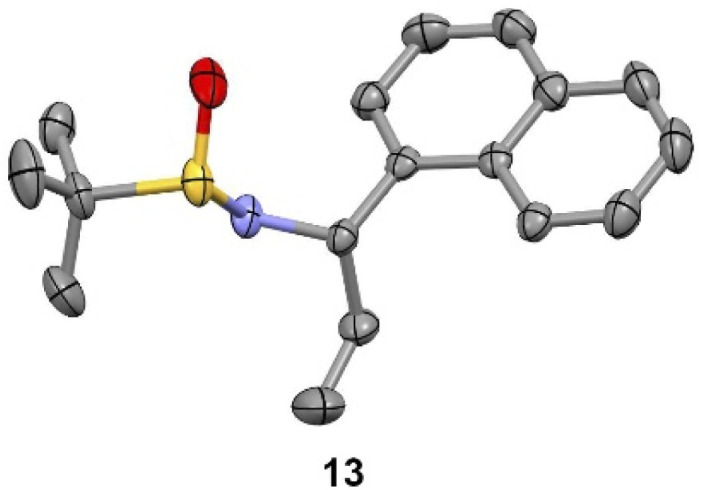
Experimental X-ray structure of 13. Thermal ellipsoids are depicted at 50%. C = grey; N = purple; S = yellow; O = red.

Now confident in our assignment, we set out to produce larger quantities of 13 (Scheme 2). Our original use of MgSO_4_ as a desiccant in the condensation reaction of 16 and 17 required multiple days to go to completion. Using Ti(OEt)_4_ instead, and conducting the reaction in CH_2_Cl_2_ at 100 °C in the microwave, provided 12 in 97% yield within 10 min. Grignard addition of EtMgBr to 12 in THF at −78 °C scaled well at multigram quantities, cleanly going to completion within 10 min, providing stereochemically pure 13 after chromatographic separation of the minor diastereoisomer (14). The sign and magnitude of the optical rotation of 13 (+114°) is consistent with what has been reported for the corresponding methyl compound (+90.7°)^15^ as well as the enantiomer of the corresponding methyl compound (−116.8°).^15^ Finally, addition of HCl in Et_2_O to a solution of 13 in Et_2_O quickly produced 5·HCl as a white solid, that could be isolated after filtration. The optical rotation of 5·HCl (+12.4) matches a literature value of +17.2.^21^ Further confirmation of the absolute configuration of 5 comes from an X-ray crystal structure of amide 18, prepared by Schotten–Baumann reaction of 5 with 2,4,6-trichlorobenzoyl chloride.^15^

**Scheme 2:**
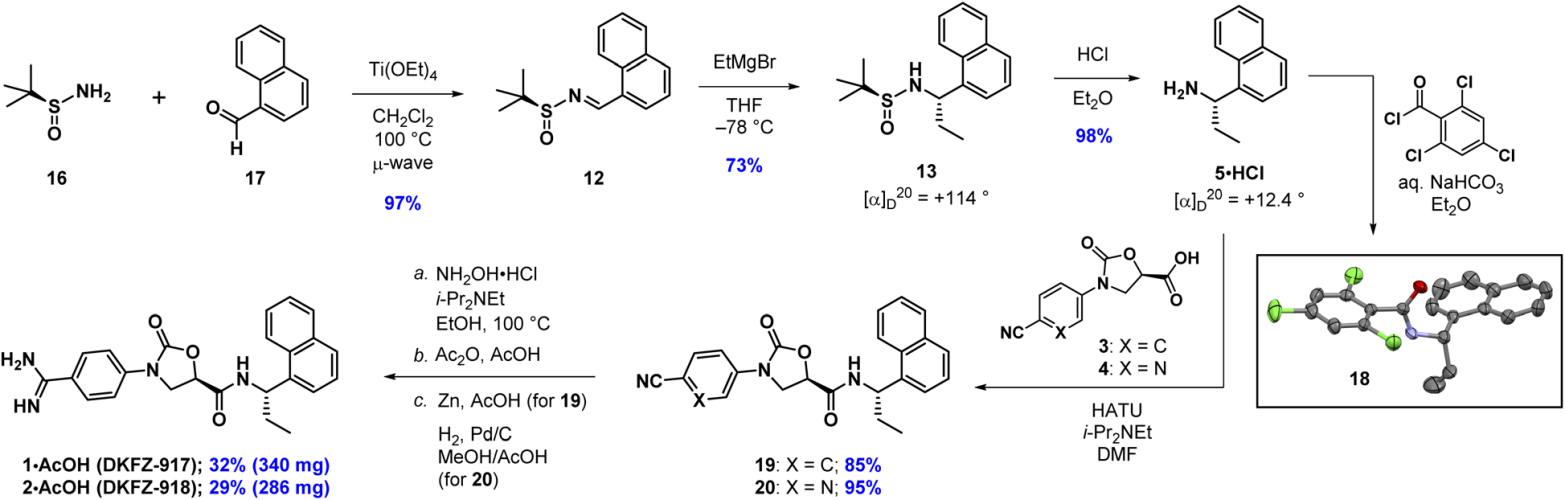
Asymmetric synthesis of 5·HCl and completion of the synthesis of DKFZ-917 and DKFZ-918. Thermal ellipsoids of 18 are depicted at 50%. C = grey; N = purple; O = red; Cl = green.

Completion of the syntheses required amide coupling, followed by conversion of the cyano group to an amidine. The former proceeded smoothly, with HATU-mediated coupling giving 19 and 20 in 85% and 95%, respectively. After numerous attempts to convert cyanides 19 and 20 into their corresponding amidines using one or two-step approaches,^22–24^ we ultimately found the following three-step protocols to be reliable and straightforward to conduct. In the event, we heated 19 and 20 with ethanolic hydroxylamine, followed by precipitation from water to give the crude *N*-hydroxyamidines. Without further purification, these were directly acetylated with acetic anhydride in acetic acid. DKFZ-917 was obtained as an acetate salt by reduction of the acetylated intermediate with zinc in acetic acid, followed by crystallization from ethanol. DKFZ-918 was best produced by hydrogenation (H_2_, Pd/C) of the corresponding acetylated intermediate at atmospheric pressure in methanol/acetic acid, followed by crystallization from ethanol. Both of these reaction sequences could be conducted on gram scale, and produced DKFZ-917 and DKFZ-918 (both with >95% purity) in 32% and 29% yields, respectively.

We were pleased to find that DKFZ-917 and DKFZ-918 behaved as expected in a KLK6 enzymatic inhibition assay with pIC_50_ values of 8.51 (8.49–8.52 95% confidence interval) and 8.22 (8.18–8.25 95% confidence interval), respectively (see ESI†). To better understand how DKFZ-917 and DKFZ-918 inhibit KLK6, we solved crystal structures of them bound to human KLK6 (Fig. 3).^25^ We found that it was critical to first co-crystallize KLK6 with the peptidic inhibitor DKFZ-878 (see ESI†), before soaking with DKFZ-917 or DKFZ-918. The 1.5 Å-resolution structure of the KLK6-DKFZ-917 complex (PDB 7QHZ) shows electron density for all atoms of DKFZ-917, and reveals the expected polar interaction between the positively charged amidine and negatively charged D189 residue in the S1 pocket. The oxazolidinone carbonyl makes indirect contact to Q192 *via* water #143, which makes hydrogen bonds to both moieties. The amide bond sits directly over active site S195, and the carbonyl forms a hydrogen bond with G193 in the oxyanion hole as well as water #1. The amide NH is engaged in a hydrogen bond to water #36. The ethyl group nicely fills the S1′ pocket and the naphthyl group extends into the S2′ pocket.

**Fig. 3:**
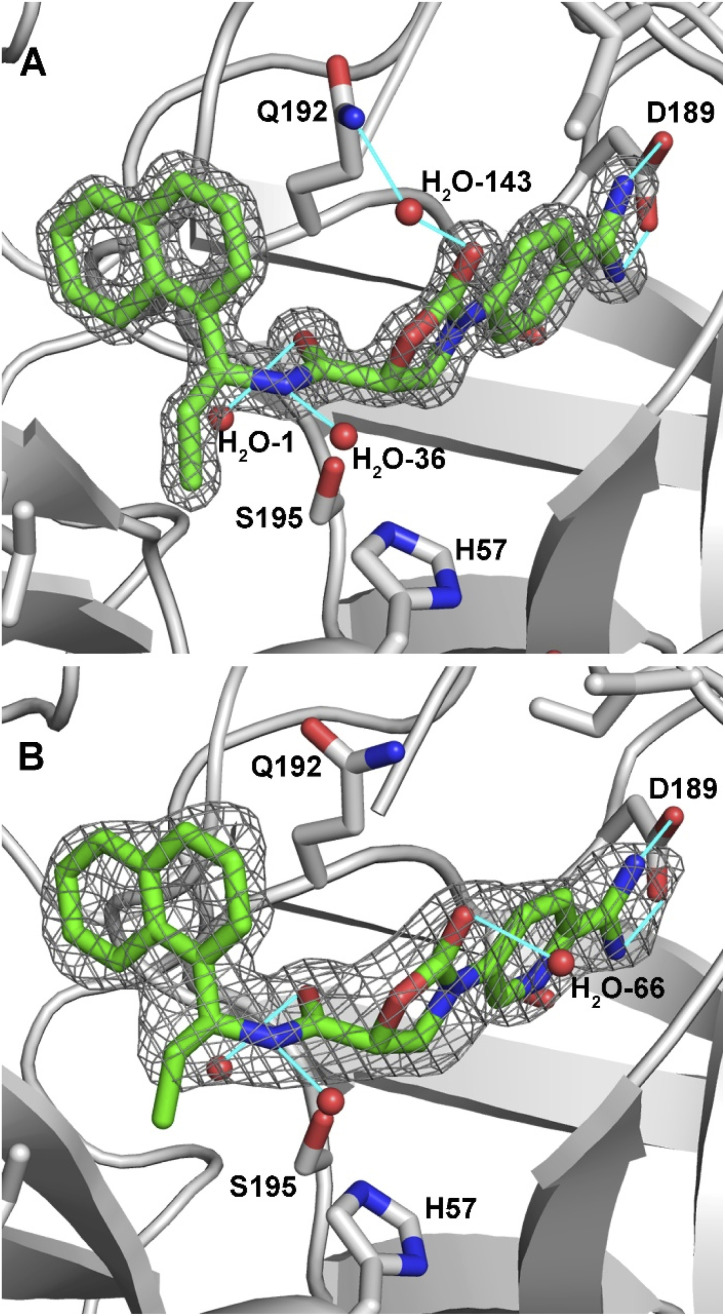
2Fo-Fc maps of inhibitors bound in the active site of KLK6. (A) DKFZ-917 (contoured at 2.0*σ*). (B) DKFZ-918 (contoured at 1.0*σ*). Selected hydrogen bonds are depicted as light blue lines.

The 1.8 Å-resolution structure of the KLK6–DKFZ-918 complex (PDB 7QI0) is very similar to that of KLK6–DKFZ-917, with the two ligands occupying virtually identical positions within the enzyme binding pocket. The largest apparent difference is the lack of a water molecule bridging the oxazolidinone carbonyl carbon to Q192. Instead, the oxazolidinone appears to make a strong hydrogen bond to water #66, which resides on the opposite face. The terminal carbon of the ethyl group shows weak electron density, potentially indicating some flexibility of this side chain. No polar interactions were observable for the pyridyl nitrogen, making its position within the binding pocket ambiguous; it has been modeled as depicted in Fig. 3 on the “inner” side of the binding pocket.

## Conclusions

In conclusion, we have established a reliable and scalable route to the two reversible and selective KLK6 inhibitors, DKFZ-917 and DKFZ-918. In doing so, we clarified with two X-ray crystal structures the unusual stereochemical outcome in the addition of ethylmagnesium bromide to an Ellman *tert*-butanesulfinimide. Furthermore, we solved crystal structures of both inhibitors bound to KLK6, revealing their binding mode. These inhibitors are now freely available to be used to investigate the role of KLK6 in varicella zoster infection, as well as in other pathological indications.

## Author contributions

A. B., D. I., and J. L. performed chemical synthesis and interpreted data. P. K. A. J. collected and interpreted protein X-ray crystallographic data. J. H. supervised the protein X-ray crystallographic work. A. K. M. conceived and organized the project, and wrote the manuscript.

## Conflicts of interest

There are no conflicts to declare.

## Supplementary Material

RA-012-D2RA04670A-s001

RA-012-D2RA04670A-s002
