# Correction: Scalable synthesis and structural characterization of reversible KLK6 inhibitors

**DOI:** 10.1039/d2ra90102a

**Published:** 2022-10-10

**Authors:** Andreas Baumann, Daniel Isak, Jasmin Lohbeck, Pravin Kumar Ankush Jagtap, Janosch Hennig, Aubry K. Miller

**Affiliations:** Cancer Drug Development Group, German Cancer Research Center (DKFZ) Im Neuenheimer Feld 280 69120 Heidelberg Germany aubry.miller@dkfz.de; Structural and Computational Biology Unit, European Molecular Biology Laboratory (EMBL) 69117 Heidelberg Germany; Chair of Biochemistry IV, Biophysical Chemistry, University of Bayreuth 95447 Bayreuth Germany; German Cancer Consortium (DKTK) Im Neuenheimer Feld 280 69120 Heidelberg Germany

## Abstract

Correction for ‘Scalable synthesis and structural characterization of reversible KLK6 inhibitors’ by Andreas Baumann *et al.*, *RSC Adv.*, 2022, **12**, 26989–26993, https://doi.org/10.1039/D2RA04670A.

The authors regret that incorrect details were given for note 20 in the original article. The correct version of note 20 is given below.

It appears as if compound **13**, or more likely its enantiomer, was prepared in ref. 18 (personal communication with A. W. H. Speed).

The Royal Society of Chemistry apologises for these errors and any consequent inconvenience to authors and readers.

## Supplementary Material

