# Supplementary material for: Scalable synthesis and structural characterization of reversible KLK6 inhibitors
Source: RSC Adv. 2022 Sep 21;12(41):26989–93. doi: 10.1039/d2ra04670a (PMC9490775; doi:10.1039/d2ra04670a)
Supplement: RA-012-D2RA04670A-s001 [file RA-012-D2RA04670A-s001.pdf]

Electronic Supplementary Material (ESI) for RSC Advances.
This journal is © The Royal Society of Chemistry 2022

Scalable Synthesis and Structural Characterization of Reversible KLK6 Inhibitors

Supporting Information

Andreas Baumann,[a] Daniel Isak,[a] Jasmin Lohbeck,[a] Pravin Kumar Ankush Jagtap,[b] Janosch Hennig,[b,c] Aubry K. Miller*,[a,d]

a. *Cancer Drug Development Group, German Cancer Research Center (DKFZ), Im Neuenheimer Feld 280, 69120, Heidelberg, Germany Address here.*

b. *Structural and Computational Biology Unit, European Molecular Biology Laboratory (EMBL), 69117 Heidelberg, Germany*

c. *Chair of Biochemistry IV, Biophysical Chemistry, University of Bayreuth, 95447 Bayreuth, Germany*

d. *German Cancer Consortium (DKTK), Im Neuenheimer Feld 280, 69120, Heidelberg, Germany*

email: aubry.miller@dkfz.de

Tel: +49 6221 42 3307

Table of Contents

Organic Synthesis 2
Methyl (4-cyanophenyl)carbamate (**7**) 3
(*R*)-4-(5-(hydroxymethyl)-2-oxooxazolidin-3-yl)benzonitrile (**8**) 3
(*R*)-3-(4-cyanophenyl)-2-oxooxazolidine-5-carboxylic acid (**3**) 4
Benzyl (6-cyanopyridin-3-yl)carbamate (**10**) 5
(*R*)-5-(5-(hydroxymethyl)-2-oxooxazolidin-3-yl)picolinonitrile (**11**) 5
(*R*)-3-(6-cyanopyridin-3-yl)-2-oxooxazolidine-5-carboxylic acid (**4**) 6
(*S*)-2-methyl-*N*-(naphthalen-1-ylmethylene)propane-2-sulfinamide (**12**) 6
(*S*)-2-methyl-*N*-[(*S*)-1-(naphthalen-1-yl)propyl]propane-2-sulfinamide (**13**) 7
(*S*)-1-(naphthalen-1-yl)propan-1-amine hydrochloride (**5**) 8
(*S*)-2,4,6-trichloro-*N*-(1-(naphthalen-1-yl)propyl)benzamide (**18**) 8
(5*R*)-3-(4-cyanophenyl)-*N*-((1*S*)-1-(naphthalen-1-yl)propyl)-2-oxooxazolidine-5-carboxamide (**19**) 9
(5*R*)-3-(6-cyanopyridin-3-yl)-*N*-((1*S*)-1-(naphthalen-1-yl)propyl)-2-oxooxazolidine-5-carboxamide (**20**) 9
(5*R*)-3-(4-carbamimidoylphenyl)-*N*-((1*S*)-1-(naphthalen-1-yl)propyl)-2-oxooxazolidine-5-carboxamide acetate(**DKFZ-917; 1**) 10
(5*R*)-3-(6-carbamimidoylpyridin-3-yl)-*N*-((1*S*)-1-(naphthalen-1-yl)propyl)-2-oxooxazolidine-5-carboxamide acetate(**DKFZ-918; 2**) 11
Small molecule x-ray crystallography 11
Protein production 13
Protein crystallography 14
Biochemical Assay 16
Literature 17

Organic Synthesis

All organic synthesis experiments were performed in solution under air with stirring unless otherwise specified. Reagent and solvents were purchased from Sigma Aldrich, Acros Organics, VWR, Roth, Merckmillipore and AlfaAesar and used without purification. Anhydrous CH_2Cl_2 and THF were prepared with an MBraun SPS800 Solvent Purification System. Anhydrous DMF was purchased from Sigma Aldrich. Thin layer chromatography (TLC) was carried out on Merck glass silica plates. TLC visualization was accomplished using 254 or 266 nm UV light charring solutions of $KMnO_4$. Flash column chromatography was performed using SiliCycle SiliaFlash® P60 (40–63 µm, 60Å particle size). Automated MPLC was performed in normal phase with a RediSep Rf system (Teledyne Isco) and RediSep Rf columns (Teledyne Isco). HPLC/MS (Agilent 1260 Infinity and an ES quadrupole Agilent 6120; column: Kinetex®2.6 µm C18 100 Å, LC Column 50 x 2.1mm; Temperature = 40 °C; Solvent A = water, 0.01% formic acid; Solvent B = acetonitrile, 0.01% formic acid; Flow Rate = 0.600mL/min; method: Gradient: 99% A →10% A [over 6 min] then 10% A→ 1% A [over 2 min]). High-resolution MS were recorded with ESI (Bruker ApexQe FT-ICR instrument), or EI (JEOL AccuTOF GCx). Optical rotations were measured on a Perkin Elmer 341 polarimeter. NMR spectra were recorded on

Bruker Avance III 9.4 T (400 MHz and 101 MHz for ^{1}H and ^{13}C, respectively) or Bruker Avance 14.1 T (600MHz and 151MHz for ^{1}H and ^{13}C, respectively) instruments at 298.1K. The residual solvent peak was used as internal reference (^{1}H NMR: $CHCl_3$ (7.26 ppm); DMSO (2.50 ppm); MeOH (3.31 ppm); ^{13}C NMR: $CHCl_3$ (77.16 ppm); DMSO (39.52 ppm); MeOH (49.00 ppm)). NMR spectra were analyzed with MestReNova: chemical shifts are reported in ppm and *J* in Hz. The following abbreviations were used to explain the multiplicities: s = singlet, d = doublet, t = triplet, q = quartet, p = pentet, m = multiplet, dt = double triplet, dd = double doublet, br s = broad singlet. Chemical names were produced with ChemDraw.

Methyl (4-cyanophenyl)carbamate (7)

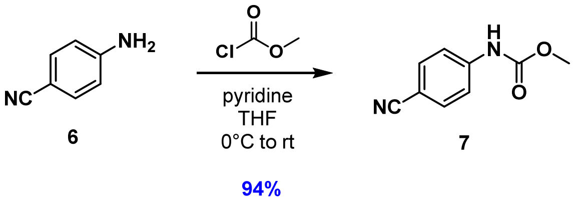

To a solution of 4-aminobenzonitrile (**5**) (1.18 g, 10.0 mmol, 1.0 eq.) in dry THF (80 mL) was added pyridine (0.97 mL, 12.0 mmol, 1.2 eq.) and methyl chloroformate (0.93 mL, 12.0 mmol, 1.2 eq.) at 0 °C under an argon.[3] The reaction mixture was allowed to warm to room temperature and stirred overnight. The reaction mixture was filtered and the filtrate was concentrated under reduced pressure. The solid residue was purified by flash column chromatography (30%→40% EtOAc in hexane) to give carbamate **7** as a white solid (1.65 g, 9.37 mmol, 94%).

TLC R_f 0.46 (60% EtOAc/hexane).

^{1}H NMR (600 MHz, $(CD_3)_2SO$) δ: 10.19 (s, 1H), 7.78–7.70 (m, 2H), 7.66–7.58 (m, 2H), 3.70 (s, 3H) ppm.

^{13}C NMR (151 MHz, $(CD_3)_2SO$) δ: 153.7, 143.6, 133.3, 119.2, 118.0, 104.1, 52.1 ppm.

LCMS t_R: 4.14 min, found 177.1.

HRMS (ESI-MS): *m/z* calcd. for $C_{18}H_{16}N_4NaO_4^+$ $[2M+Na]^+$: 375.1064, found 375.1063.

(*R*)-4-(5-(hydroxymethyl)-2-oxooxazolidin-3-yl)benzonitrile (**8**)

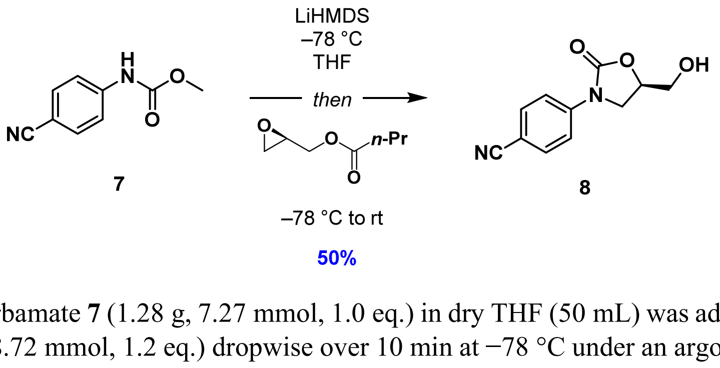

To a solution of carbamate **7** (1.28 g, 7.27 mmol, 1.0 eq.) in dry THF (50 mL) was added LiHMDS (1 M in THF, 8.72 mL, 8.72 mmol, 1.2 eq.) dropwise over 10 min at −78 °C under an argon atmosphere. The solution turned dark red and was stirred for 1 h before (*R*)-(–)-glycidyl butyrate (1.13 mL, 8.00 mmol, 1.1 eq.) was added dropwise. After 1 h at −78 °C, the reaction mixture was allowed to warm to room temperature and stirred overnight. The reaction mixture was quenched with saturated aqueous NH_4Cl

(50 mL) and diluted with EtOAc (50 mL). The layers were separated and the aqueous phase was extracted with EtOAc (2 × 150 mL). The combined organic layers were washed with brine (50 mL), dried ($MgSO_4$), filtered, and concentrated under reduced pressure. The mixture was crystallized from hot EtOAc/hexane to give oxazolidinone **8** as a beige solid (789 mg, 3.62 mmol, 50%).

TLC R_f 0.2 (5% MeOH/DCM).

^{1}H NMR (600 MHz, $(CD_3)_2SO$) δ: 7.87–7.83 (m, 2H), 7.78–7.74 (m, 2H), 5.25 (t, *J* = 5.6 Hz, 1H), 4.75 (ddt, *J* = 9.4, 6.0, 3.6 Hz, 1H), 4.13 (t, *J* = 9.0 Hz, 1H), 3.87 (dd, *J* = 9.0, 6.0 Hz, 1H), 3.69 (ddd, *J* = 12.4, 5.6, 3.2 Hz, 1H), 3.57 (ddd, *J* = 12.4, 5.8, 3.9 Hz, 1H) ppm.

^{13}C NMR (151 MHz, $(CD_3)_2SO$) δ: 154.3, 142.6, 133.3, 119.0, 117.7, 105.0, 73.6, 61.6, 45.8 ppm.

LCMS t_R: 3.46 min, found 219.1.

HRMS (ESI-MS): *m/z* calcd. for $C_{13}H_{10}F_3N_2O_5^-$ [M+TFA−H]$^-$: 331.0547, found 331.0540.

(*R*)-3-(4-cyanophenyl)-2-oxooxazolidine-5-carboxylic acid (**3**)

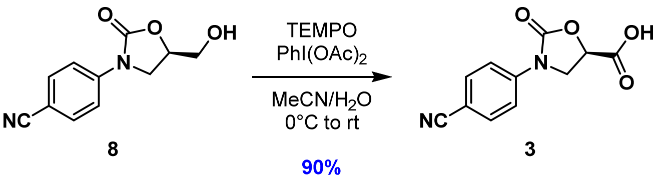

To a solution of alcohol **8** (772 mg, 3.54 mmol, 1.0 eq.) in a 1:1 mixture of MeCN/water (20 mL) was added (diacetoxyiodo)benzene (2.85 g, 8.85 mmol, 2.5 eq.) followed by TEMPO (111 mg, 708 µmol, 0.2 eq.) at 0 °C. After 30 min, the reaction mixture was warmed to room temperature. After 6 d, it was diluted with EtOAc (50 mL) and saturated aqueous $NaHCO_3$ (50 mL). The layers were separated and the organic layer was extracted with $NaHCO_3$ (50 mL). The combined aqueous phases were acidified with 3 M HCl and then extracted with EtOAc (2 × 100 mL). The combined organic layers were dried ($MgSO_4$), filtered, and concentrated under reduced pressure to give acid **3** as a light yellow solid (744 mg, 3.20 mmol, 90%), which was used in the next step without further purification.

TLC R_f 0.3 (15% MeOH/DCM).

^{1}H NMR (600 MHz, $(CD_3)_2SO$) δ: 7.88–7.83 (m, 2H), 7.78–7.73 (m, 2H), 5.24 (dd, *J* = 9.8, 5.3 Hz, 1H), 4.40 (t, *J* = 9.6 Hz, 1H), 4.18 (dd, *J* = 9.4, 5.3 Hz, 1H) ppm.

^{13}C NMR (151 MHz, $(CD_3)_2SO$) δ: 170.0, 153.5, 142.0, 133.3, 118.9, 118.1, 105.6, 69.3, 47.2 ppm.

HRMS (ESI-MS): *m/z* calcd. for $C_{22}H_{15}N_4O_8^-$ [2M−H]$^-$: 463.0895, found 463.0893.

Benzyl (6-cyanopyridin-3-yl)carbamate (**10**)

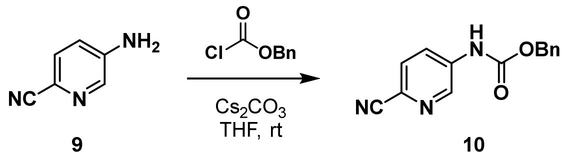

9 **10**

91%

To a suspension of 5-aminopyridine-2-carbontitrile (**9**) (1.0 g, 8.40 mmol, 1.0 eq.) and Cs_2CO_3 (4.11 g, 12.6 mmol, 1.5 eq.) in dry THF (80 mL) was added benzyl chloroformate (1.8 mL, 12.6 mmol, 1.5 eq.) under an argon atmosphere.[4] The resulting mixture was stirred at room temperature for 2 d. TLC indicated that the reaction mixture still contained starting material, so further benzyl chloroformate (0.6 mL, 4.20 mmol, 0.5 eq.) was added. After stirring for another 20 h, the reaction mixture was diluted with EtOAc (150 mL) and washed with water (100 mL). The layers were separated and the aqueous phase was extracted with EtOAc (2 × 150 mL). The combined organic layers were washed with brine (150 mL), dried ($MgSO_4$), filtered, and concentrated under reduced pressure. The obtained yellow solid was washed excessively with hexane to give carbamate **10** as a light orange solid (1.94 g, 7.66 mmol, 91%).

TLC R_f 0.7 (50% EtOAc/hexane).

^{1}H NMR (600 MHz, CD_3OD) δ: 8.69 (d, J = 2.6 Hz, 1H), 8.18 (dd, J = 8.6, 2.6 Hz, 1H), 7.78 (d, J = 8.6 Hz, 1H), 7.44–7.41 (m, 2H), 7.40–7.30 (m, 3H), 5.22 (s, 2H) ppm.

^{13}C NMR (151 MHz, CD_3OD) δ: 155.1, 142.2, 141.0, 137.5, 130.5, 129.6, 129.4, 129.3, 127.3, 126.0, 118.4, 67.4 ppm.

LCMS t_R: 4.90 min, found 254.1.

HRMS (ESI-MS): *m/z* calcd. for $C_{28}H_{22}N_6NaO_4^+$ $[2M+Na]^+$: 529.1595, found 529.1593.

(*R*)-5-(5-(hydroxymethyl)-2-oxooxazolidin-3-yl)picolinonitrile (**11**)

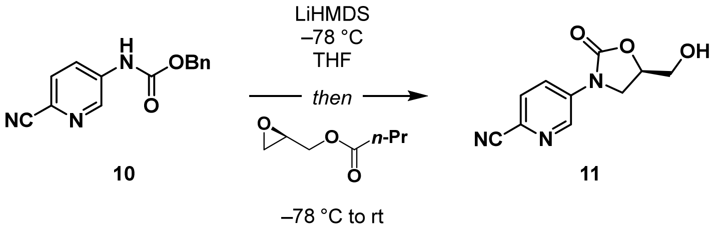

10 **11**

44%

To a solution of carbamate **10** (1.92 g, 7.58 mmol, 1.0 eq.) in anhydrous THF (50 mL) was added LiHMDS (1 M in THF, 9.10 mL, 9.10 mmol, 1.2 eq.) dropwise over 10 min at −78 °C under an argon atmosphere. The solution turned dark red and was stirred for 1 h before (*R*)-(–)-glycidyl butyrate (1.18 mL, 8.34 mmol, 1.1 eq.) was added dropwise. Ater 1 h at −78 °C, the reaction mixture was allowed to warm to room temperature and stirred overnight. The reaction mixture was quenched with saturated aqueous NH_4Cl (50 mL) and diluted with EtOAc (50 mL). The layers were separated and the aqueous phase was extracted EtOAc (2 × 150 mL). The combined organic layers were washed with brine (50 mL), dried ($MgSO_4$), filtered, and concentrated under reduced pressure. The mixture was recrystallized from hot EtOAc/hexane to give oxazolidinone **11** as a beige solid (723 mg, 3.34 mmol, 44%).

TLC R_f 0.3 (5% MeOH/DCM).

^{1}H NMR (600 MHz, $(CD_3)_2SO$) δ: 8.95 (dd, J = 2.7, 0.7 Hz, 1H), 8.21 (dd, J = 8.7, 2.7 Hz, 1H), 8.05 (dd, J = 8.8, 0.7 Hz, 1H), 5.27 (t, J = 5.6 Hz, 1H), 4.81 (ddt, J = 9.3, 5.9, 3.6 Hz, 1H), 4.18 (t, J = 9.0 Hz, 1H), 3.93 (dd, J = 9.0, 5.9 Hz, 1H), 3.70 (ddd, J = 12.5, 5.5, 3.3 Hz, 1H), 3.59 (ddd, J = 12.4, 5.7, 3.9 Hz, 1H) ppm.

^{13}C NMR (151 MHz, $(CD_3)_2SO$) δ: 154.4, 140.2, 138.3, 129.3, 125.9, 124.5, 117.7, 74.3, 61.5, 45.4 ppm.

LCMS t_R: 2.69 min, found 220.1.

HRMS (ESI-MS): m/z calcd. for $C_{12}H_9F_3N_3O_5^-$ [M+TFA−H]$^-$: 332.0500, found 332.0493.

(R)-3-(6-cyanopyridin-3-yl)-2-oxooxazolidine-5-carboxylic acid (**4**)

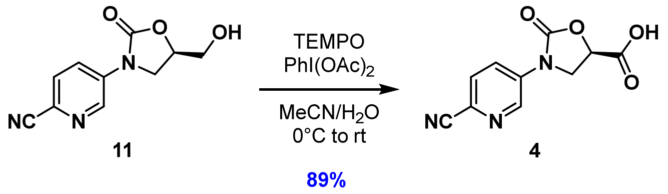

To a solution of alcohol **11** (701 mg, 3.20 mmol, 1.0 eq.) in a 1:1 mixture of MeCN/water (20 mL) was added (diacetoxyiodo)benzene (2.58 g, 8.00 mmol, 2.5 eq.) followed by TEMPO (100 mg, 640 µmol, 0.2 eq.) at 0 °C. After 30 min, the reaction mixture was warmed to room temperature. After 6 d, it was diluted with EtOAc (50 mL) and saturated aqueous $NaHCO_3$ (50 mL). The layers were separated and the organic layer was extracted with $NaHCO_3$ (50 mL). The combined aqueous phases were acidified with 3 M HCl and then extracted with EtOAc (2 × 100 mL). The combined organic layers were dried ($MgSO_4$), filtered, and concentrated under reduced pressure to give acid **4** as a light yellow solid (662 mg, 2.84 mmol, 89%), which was used in the next step without further purification.

TLC R_f 0.5 (10% MeOH/DCM).

^{1}H NMR (600 MHz, $(CD_3)_2SO$) δ: 8.94 (dd, J = 2.7 Hz, 1H), 8.22 (dd, J = 8.7, 2.7 Hz, 1H), 8.07 (d, J = 8.7 Hz, 1H), 5.30 (dd, J = 9.8, 5.4 Hz, 1H), 4.44 (t, J = 9.6 Hz, 1H), 4.26 (dd, J = 9.3, 5.4 Hz, 1H) ppm.

^{13}C NMR (151 MHz, $(CD_3)_2SO$) δ: 169.8, 153.6, 140.6, 137.8, 129.3, 126.4, 125.1, 117.6, 69.8, 46.7 ppm.

HRMS (ESI-MS): m/z calcd. for $C_{20}H_{13}N_6O_8^-$ [2M−H]$^-$: 465.0800, found 465.0801.

(S)-2-methyl-N-(naphthalen-1-ylmethylene)propane-2-sulfinamide (**12**)

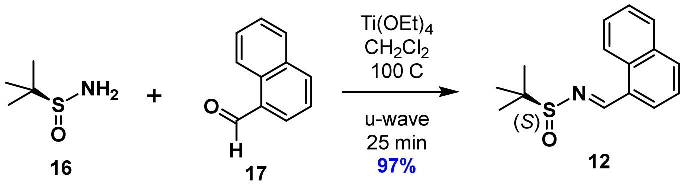

In a microwave vial 1-naphthaldehyde (**17**) (1.4 mL, 10.3 mmol, 1.2 eq.) was dissolved in dry dichloromethane (5 mL) under an argon atmosphere. S-(–)-2-methylpropane-2-sulfinamide (**16**) (1.05 g, 8.66 mmol, 1.0 eq.) and titanium(IV) ethoxide (4.0 mL, 19.1 mmol, 2.2 eq.) were added and the vial was rinsed with dry dichloromethane (9 mL).[1,2] The reaction mixture was heated in the microwave for

25 min at 100 °C. Afterwards it was quenched with water (300 mL), diluted with dichloromethane (100 mL) and filtered over a pad of celite. The aqueous phase was extracted with dichloromethane (3 x 50 mL). The combined organic layers were dried ($MgSO_4$), filtered, and concentrated under reduced pressure. The product was purified by flash column chromatography (20% EtOAc in hexane) to give aldimine **12** as a yellow solid (2.17 g, 8.37 mmol, 97%).

TLC R_f 0.33 (20% EtOAc/hexane).

^{1}H NMR (600 MHz, CD_3OD) δ: 9.09 (s, 1H), 9.01 (dt, *J* = 8.6, 1.0 Hz, 1H), 8.10 (d, *J* = 8.1 Hz, 1H), 8.06 (dd, *J* = 7.2, 1.3 Hz, 1H), 8.00–7.96 (m, 1H), 7.68–7.57 (m, 3H), 4.88 (s, 1H), 1.31 (s, 9H) ppm.

^{13}C NMR (101 MHz, CD_3OD) δ: 164.4, 135.5, 134.9, 133.6, 132.4, 130.4, 130.1, 129.3, 127.7, 126.4, 125.1, 58.8, 22.7 ppm.

LCMS t_R: 5.48 min, found 260.1.

HRMS (ESI-MS): *m/z* calcd. for $C_{30}H_{34}N_2NaO_2S_2^+$ $[2M+Na]^+$: 541.1954, found 541.1953.

(*S*)-2-methyl-*N*-[(*S*)-1-(naphthalen-1-yl)propyl]propane-2-sulfinamide (**13**)

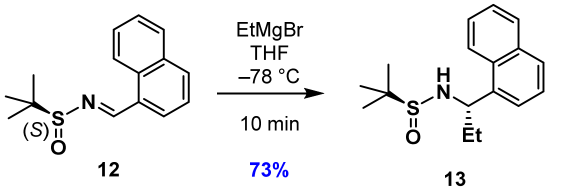

To a solution of *tert*-butanesulfinyl aldimine **12** (2.84 g, 11.0 mmol, 1.0 eq.) in dry THF (60 mL) was added a solution of EtMgBr (3 M in Et_2O, 9.14 mL, 27.4 mmol, 2.5 eq) dropwise over 5 min at −78 °C under argon. The reaction mixture was stirred for 10 min before it was quenched with sat. aq. NH_4Cl (100 mL) and diluted with EtOAc (50 mL). The layers were separated and the aqueous phase was extracted with EtOAc (2 x 50 mL). The combined organic layers were dried ($MgSO_4$), filtered, and concentrated under reduced pressure. The crude product, a mixture of diastereomers (10:1), was purified by flash column chromatography (40% EtOAc in hexane) to give **13** as a colorless solid (2.34 g, 8.08 mmol, 73%). X-ray quality crystals were obtained via the vapor diffusion method from Et_2o/pentane.

TLC R_f 0.46 (60% EtOAc/hexane).

$[\alpha]_D^{20}$ = + 114 (c = 1.21, $CHCl_3$).

^{1}H NMR (400 MHz, $CDCl_3$) δ: 8.23 (d, *J* = 9.1 Hz, 1H), 7.89–7.85 (m, 1H), 7.80 (d, *J* = 8.2 Hz, 1H), 7.58–7.44 (m, 4H), 5.21–5.11 (m, 1H), 3.57 (d, *J* = 3.0 Hz, 1H), 2.24–2.07 (m, 2H), 1.23 (s, 9H), 0.84 (t, *J* = 7.4 Hz, 3H) ppm.

^{13}C NMR (101 MHz, $CDCl_3$) δ: 137.5, 134.2, 131.2, 129.1, 128.5, 126.4, 125.8, 125.4, 124.7, 123.4, 55.8, 28.6, 22.8, 10.3 ppm.

LCMS t_R: 5.24 min, found 290.2.

HRMS (ESI-MS): *m/z* calcd. for $C_{34}H_{46}N_2NaO_2S_2^+$ $[2M+Na]^+$: 601.2893, found 601.2891.

(S)-1-(naphthalen-1-yl)propan-1-amine hydrochloride (**5**)

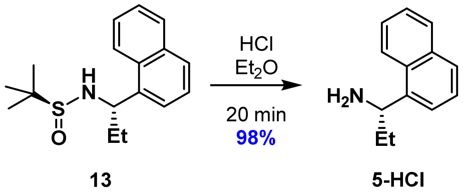

To a solution of *tert*-butanesulfinamide **13** (2.23 g, 7.70 mmol, 1.0 eq.) in Et_2O (50 mL) was added a solution of HCl in Et_2O (4 M, 11.6 mL, 23.2 mmol, 3.0 eq.) dropwise at 0 °C. After 5 min, the ice bath was removed and the reaction mixture stirred for 15 min at room temperature. The resulting white precipitate was filtered, washed excessively with Et_2O, and dried under high vacuum to give the HCl salt of amine **5** as a white solid (1.67 g, 7.53 mmol, 98%).

$[\alpha]_D^{20}$ = + 12.4 (c = 1.39, CD_3OD).

^{1}H NMR (400 MHz, CD_3OD) δ: 8.20 (d, J = 8.3 Hz, 1H), 7.99–7.94 (m, 2H), 7.71 (dd, J = 7.3, 1.2 Hz, 1H), 7.67–7.55 (m, 3H), 5.22 (dd, J = 8.1, 6.4 Hz, 1H), 2.31–2.06 (m, 2H), 0.95 (t, J = 7.5 Hz, 3H) ppm.

^{13}C NMR (101 MHz, CD_3OD) δ: 135.4, 134.4, 132.4, 130.6, 130.2, 128.2, 127.4, 126.5, 124.6, 123.3, 52.5, 29.3, 10.5 ppm.

LCMS t_R: 3.19 min, found 169.2.

HRMS (EI-MS): *m/z* calcd. for $C_{13}H_{15}N^{\bullet+}$ $[M]^{\bullet+}$: 185.1199, found 185.1214.

(S)-2,4,6-trichloro-N-(1-(naphthalen-1-yl)propyl)benzamide (**18**)

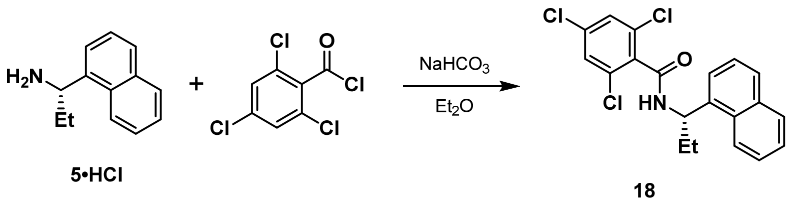

In a 50 mL separation funnel amine **5**•HCl (30 mg, 135 µmol, 1 eq.) was dissolved in Et_2O (10 mL) and a saturated solution of $NaHCO_3$ (10 mL). Trichlorobenzoylchloride (25.4 µL, 162 µmol, 1.2 eq.) was added and the separation funnel shaken for 3 min. The layers were separated, and the aqueous layer was extracted with EtOAc (2 x 10 mL). The combined organic layers were dried ($MgSO_4$), filtered, and concentrated under reduced pressure. Crystals of **18** suitable for x-ray crystallography were obtained by vapor diffusion (Et_2O/pentane).

R_f: 0.9 (60% EtOAc/ hexane).

LC-MS: $[M+H^+]$ 394.0

(5*R*)-3-(4-cyanophenyl)-*N*-((1*S*)-1-(naphthalen-1-yl)propyl)-2-oxooxazolidine-5-carboxamide (**19**)

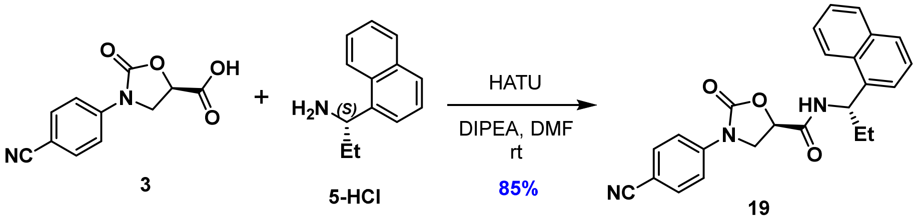

To a solution of acid **3** (690 mg, 2.97 mmol, 1.0 eq.) and amine **5**•HCl (659 mg, 2.97 mmol, 1.0 eq.) in dry DMF (30 mL) were added HATU (1.36 g, 3.56 mmol, 1.2 eq.) and *i*-Pr_2NEt (1.52 mL, 8.91 mmol, 3.0 eq.) at 0 °C under an argon atmosphere. After the addition, the reaction mixture was allowed to warm to room temperature. After 16 h, the reaction mixture was diluted with EtOAc (50 mL) and washed with brine (100 mL). The aqueous phase was extracted with EtOAc (2 × 100 mL) and the combined organic layers were dried ($MgSO_4$), filtered, and concentrated under reduced pressure. The product was purified by flash column chromatography (30%→50% EtOAc in hexane) to give amide **19** as a colorless solid (1.01 g, 2.53 mmol, 85%).

TLC R_f 0.5 (60% EtOAc/hexane).

^{1}H NMR (600 MHz, $(CD_3)_2SO$) δ: 9.08 (d, *J* = 8.2 Hz, 1H), 8.15 (d, *J* = 8.4 Hz, 1H), 7.94 (dd, *J* = 8.1, 1.5 Hz, 1H), 7.89–7.79 (m, 3H), 7.79–7.73 (m, 2H), 7.60–7.47 (m, 4H), 5.61–5.51 (m, 1H), 5.21 (dd, *J* = 9.3, 5.7 Hz, 1H), 4.37 (t, *J* = 9.3 Hz, 1H), 4.07–4.01 (m, 1H), 1.96–1.82 (m, 2H), 0.98 (t, *J* = 7.3 Hz, 3H) ppm.

^{13}C NMR (151 MHz, $(CD_3)_2SO$) δ: 167.3, 153.5, 142.0, 138.7, 133.4, 133.3, 130.5, 128.7, 127.5, 126.3, 125.7, 125.5, 122.99, 122.95, 118.9, 118.0, 105.4, 70.6, 50.3, 47.7, 28.5, 11.3 ppm.

LCMS t_R: 5.45 min, found 400.1.

HRMS (ESI-MS): *m/z* calcd. for $C_{26}H_{21}F_3N_3O_5^-$ $[M+TFA-H]^-$: 512.1439, found 512.1438.

(5*R*)-3-(6-cyanopyridin-3-yl)-*N*-((1*S*)-1-(naphthalen-1-yl)propyl)-2-oxooxazolidine-5-carboxamide (**20**)

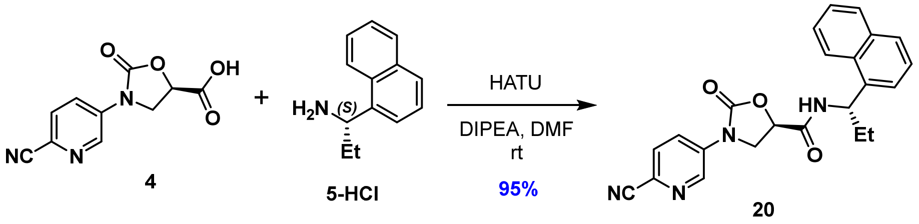

To a solution of acid **4** (627 mg, 2.69 mmol, 1.0 eq.) and amine **5**•HCl (596 mg, 2.69 mmol, 1.0 eq.) in dry DMF (30 mL) were added HATU (1.23 g, 3.23 mmol, 1.2 eq.) .) and *i*-Pr_2NEt (1.37 mL, 8.07 mmol, 3.0 eq.) at 0 °C under an argon atmosphere. After the addition, the reaction mixture was allowed to warm to room temperature. After 16 h, the reaction mixture was diluted with EtOAc (50 mL) and washed with brine (100 mL). The aqueous phase was extracted with EtOAc (2 × 100 mL) and the combined organic layers were dried ($MgSO_4$), filtered, and concentrated under reduced pressure. The product was purified by flash column chromatography (50%→60% EtOAc in hexane) to give amide **20** as a light yellow solid (1.17 g contained EtOAc according to NMR, calculated yield: 1.03 g, 2.56 mmol, 95%).

TLC R_f 0.6 (75% EtOAc/hexane).

^{1}H NMR (600 MHz, $(CD_3)_2SO$) δ: 9.09 (d, *J* = 8.3 Hz, 1H), 8.96 (d, *J* = 2.7 Hz, 1H), 8.21 (dd, *J* = 8.7, 2.7 Hz, 1H), 8.15 (d, *J* = 8.4 Hz, 1H), 8.06 (d, *J* = 8.7 Hz, 1H), 7.94 (dd, *J* = 8.1, 1.4 Hz, 1H), 7.84 (d, *J* = 8.1 Hz, 1H), 7.59–7.47 (m, 4H), 5.59–5.54 (m, 1H), 5.26 (dd, *J* = 9.3, 5.7 Hz, 1H), 4.41 (t, *J* = 9.3 Hz, 1H), 4.12 (dd, *J* = 9.2, 5.7 Hz, 1H), 1.96–1.83 (m, 2H), 0.98 (t, *J* = 7.3 Hz, 3H) ppm.

^{13}C NMR (151 MHz, $(CD_3)_2SO$) δ: 167.0, 153.7, 140.5, 138.7, 137.8, 133.4, 130.5, 129.3, 128.8, 127.5, 126.3, 125.7, 125.5, 124.9, 123.0, 123.0, 117.6, 71.1, 50.3, 47.1, 28.5, 11.3 ppm.

LCMS t_R: 5.30 min, found 401.2.

HRMS (ESI-MS): *m/z* calcd. for $C_{46}H_{40}N_8NaO_6^+$ $[2M+Na]^+$: 823.2963, found 823.2964.

(5*R*)-3-(4-carbamimidoylphenyl)-*N*-((1*S*)-1-(naphthalen-1-yl)propyl)-2-oxooxazolidine-5-carboxamide acetate(**DKFZ-917; 1**)

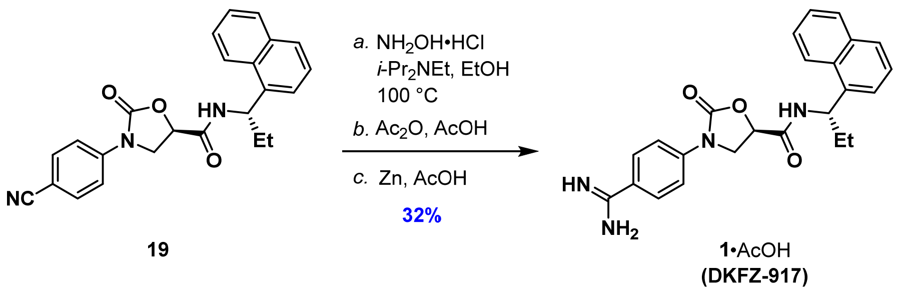

A solution of amide **19** (882 mg, 2.21 mmol, 1.0 eq.), $NH_2OH{\cdot}HCl$ (764 mg, 11.0 mmol, 5.0 eq.), and *i*-Pr_2NEt (1.81 ml, 11.0 mmol, 5.0 eq.) in EtOH (70 mL) was heated to 100 °C for 20 h in a sealed glass reactor. Subsequently, the solvent was almost removed completely under reduced pressure and the remaining concentrated solution was dripped into a Büchner funnel filled with water. The obtained precipitate was filtered and the colorless solid was co-evaporated with toluene (3 × 100 mL) and dried under high vacuum overnight. This solid was then dissolved in AcOH (25 mL) and Ac_2O (0.84 mL, 8.84 mmol, 4.0 eq) was added. The reaction mixture was stirred for 90 min and was then quenched by the addition of water (2 mL). The solvent was removed under reduced pressure, the crude solid was co-evaporated with toluene (3 × 100 mL), dried under high vacuum overnight, and purified via MPLC (80 g column, 0%→8.5% MeOH in CH_2Cl_2). The obtained colorless solid was dissolved in AcOH (30 mL) and zinc dust (1.44 g, 22.1 mmol, 10 eq) was added. The reaction mixture was stirred for 2 h and then filtered over a pad of celite, which was then rinsed with AcOH (ca. 100 mL). The mixture was concentrated under reduced pressure and crystallized from a minimal amount of hot EtOH to give the acetate salt of amidine **1** (**DKFZ-917**) as a colorless solid (340 mg, 0.713 mmol, 32% over three steps).

^{1}H NMR (600 MHz, CD_3OD) δ: 8.14 (d, *J* = 8.5 Hz, 1H), 7.88–7.84 (m, 1H), 7.83–7.73 (m, 5H), 7.57–7.52 (m, 1H), 7.52–7.48 (m, 1H), 7.48–7.41 (m, 2H), 5.71 (dd, *J* = 9.0, 5.8 Hz, 1H), 5.21 (dt, *J* = 10.2, 3.9 Hz, 1H), 4.37 (t, *J* = 8.9 Hz, 1H), 4.13 (dd, *J* = 9.5, 5.7 Hz, 1H), 2.11–1.96 (m, 2H), 1.91 (s, AcOH, 3H), 1.06 (t, *J* = 7.4 Hz, 3H) ppm.

^{13}C NMR (151 MHz, CD_3OD) δ: 176.4 (AcOH), 169.9, 167.6, 155.3, 144.4, 139.1, 135.4, 132.4, 130.01, 129.96, 129.0, 127.7, 126.7, 126.4, 124.3, 124.1, 123.9, 119.2, 72.3, 52.5, 49.0 (observed by HSQC), 29.6, 24.0 (AcOH), 11.7 ppm.

LCMS t_R: 3.95 min, found 417.2.

HRMS (ESI-MS): *m/z* calcd. for $C_{24}H_{25}N_4O_3^+$ $[M+H]^+$: 417.1921, found 417.1920.

(5*R*)-3-(6-carbamimidoylpyridin-3-yl)-*N*-((1*S*)-1-(naphthalen-1-yl)propyl)-2-oxooxazolidine-5-carboxamide acetate(**DKFZ-918; 2**)

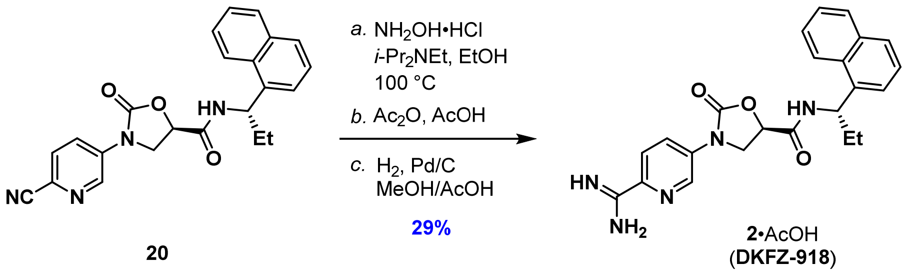

A solution of amide **20** (827 mg, 2.07 mmol, 1.0 eq.), $NH_2OH{\cdot}HCl$ (723 mg, 10.4 mmol, 5.0 eq.), and *i*-Pr_2NEt (1.71 ml, 10.4 mmol, 5.0 eq.) in EtOH (70 mL) was heated to 100 °C in a sealed glass reactor for 2 h. The solvent was almost removed completely under reduced pressure and the remaining suspension was filtered and the residual solid was excessively washed with water and then co-evaporated with toluene (3 × 100 mL) and dried under high vacuum overnight. This was then dissolved in AcOH (20 mL) and Ac_2O (0.78 mL, 8.28 mmol, 4.0 eq) was added. The reaction mixture was stirred for 1 h and was then quenched by the addition of water (2 mL). The solvent was removed under reduced pressure and the crude solid was co-evaporated with toluene (3 × 100 mL) and dried under high vacuum overnight. The obtained colorless solid was dissolved in AcOH/MeOH (1:1, 60 mL) and Pd/C was added under argon atmosphere. The flask was then purged with hydrogen three times and vigorously stirred. After 22 h, the reaction mixture was filtered over a plug of celite, concentrated under reduced pressure, crystallized from a minimal amount of EtOH, to give the acetate salt of amidine **2** (**DKFZ-918**) as a grey solid (286 mg, 0.599 mmol, 29% over three steps).

^{1}H NMR (600 MHz, $(CD_3)_2SO$) δ: 9.15 (d, *J* = 8.2 Hz, 1H), 9.00 (d, *J* = 2.5 Hz, 1H), 8.26–8.18 (m, 2H), 8.16 (d, *J* = 8.5 Hz, 1H), 7.94 (dd, *J* = 8.0, 1.5 Hz, 1H), 7.84 (d, *J* = 8.1 Hz, 1H), 7.60–7.54 (m, 2H), 7.54–7.49 (m, 2H), 5.61–5.53 (m, 1H), 5.28 (dd, *J* = 9.3, 5.8 Hz, 1H), 4.43 (t, *J* = 9.2 Hz, 1H), 4.14 (dd, *J* = 9.2, 5.8 Hz, 1H), 1.91 (dq, *J* = 10.5, 6.8 Hz, 2H), 1.75 (s, AcOH, 3H), 0.98 (t, *J* = 7.3 Hz, 3H) ppm.

^{13}C NMR (151 MHz, $(CD_3)_2SO$) δ: 175.9 (AcOH), 167.1, 161.6, 153.7, 139.9, 138.7, 138.6, 137.8, 133.4, 130.5, 128.8, 127.5, 126.3, 125.7, 125.5, 125.2, 123.1, 123.0, 122.7, 71.1, 50.3, 47.2, 28.5, 24.3 (AcOH), 11.4 ppm.

LCMS t_R: 4.01 min, found 418.2.

HRMS (ESI-MS): *m/z* calcd. for $C_{23}H_{24}N_5O_3^+$ $[M+H]^+$: 418.1874, found 418.1873.

Small molecule x-ray crystallography

13: colourless crystal (brick), dimensions 0.145 x 0.052 x 0.035 mm^3, T=200(2) K, radiation MoKα, lambda=0.71073 Å, 0.5 deg omega-scans with CCD area detector, covering the asymmetric unit in reciprocal space with a mean redundancy of 8.44and a completeness of 100.0% to a resolution of 0.84 Å, intensities were corrected for Lorentz and polarization effects, an empirical scaling and absorption correction was applied using SADABS[4] based on the Laue symmetry of the reciprocal space, mu=0.19mm^{-1}, structure solved with SHELXT-2018/2[5] and refined against F^2 with a Full-matrix least-squares algorithm using the SHELXL-2018/3 software,[6] hydrogen atoms were treated using appropriate riding models.

Crystal data and structure refinement

Identification code	**13 (**CCDC 2130651)
Empirical formula	$C_{17}H_{23}NOS$
Formula weight	289.42
Temperature	200(2) K
Wavelength	0.71073 Å
Crystal system	monoclinic
Space group	$P2_1$
Z	2
Unit cell dimensions	a = 7.5133(4) Å α = 90 deg.
	b = 9.5794(4) Å β = 105.1664(12) deg.
	c = 12.1165(6) Å γ = 90 deg.
Volume	841.69(7) $Å^3$
Density (calculated)	1.14 g/cm^3
Absorption coefficient	0.19 mm^{-1}
Crystal shape	brick
Crystal size	0.145 x 0.052 x 0.035 mm^3
Crystal colour	colourless
Theta range for data collection	1.7 to 25.0 deg.
Index ranges	-8≤h≤8, -11≤k≤11, -14≤l≤14
Reflections collected	13381
Independent reflections	2974 (R(int) = 0.0330)
Observed reflections	2662 (I > 2σ(I))
Absorption correction	Semi-empirical from equivalents
Max. and min. transmission	0.96 and 0.93
Refinement method	Full-matrix least-squares on F^2
Data/restraints/parameters	2974 / 1 / 185
Goodness-of-fit on F^2	1.06
Final R indices (I>2sigma(I))	R1 = 0.032, wR2 = 0.069
Absolute structure parameter	0.07(3)
Largest diff. peak and hole	0.17 and -0.18 $eÅ^{-3}$

18: colourless crystal (brick), dimensions 0.106 x 0.088 x 0.047 mm^3, T=200(2) K, radiation MoKα, lambda=0.71073 Å, 0.5 deg omega-scans with CCD area detector, covering the asymmetric unit in reciprocal space with a mean redundancy of 5.4 and a completeness of 100.0% to a resolution of 0.79 Å, intensities were corrected for Lorentz and polarization effects, an empirical scaling and absorption correction was applied using SADABS[4] based on the Laue symmetry of the reciprocal space, mu=0.49mm^{-1}, structure solved with SHELXT-2014[5] and refined against F^2 with a Full-matrix least-squares algorithm using the SHELXL-2018/3 software,[6] hydrogen atoms were treated using appropriate riding models.

Crystal data and structure refinement

Identification code	**18 (**CCDC 2130650)
Empirical formula	$C_{20}H_{16}Cl_3NO$
Formula weight	392.69
Temperature	200(2) K
Wavelength	0.71073 Å
Crystal system	trigonal
Space group	$P3_1$
Z	6

Unit cell dimensions	a =	11.8686(1) Å	α =	90 deg.
	b =	11.8686(1) Å	β =	90 deg.
	c =	23.1699(3) Å	γ =	120 deg.
Volume	2826.53(5) $Å^3$			
Density (calculated)	1.38 g/cm^3			
Absorption coefficient	0.49 mm^{-1}			
Crystal shape	brick			
Crystal size	0.106 x 0.088 x 0.047 mm^3			
Crystal colour	colourless			
Theta range for data collection	0.9 to 26.7 deg.			
Index ranges	$-14 \le h \le 14$, $-14 \le k \le 14$, $-29 \le l \le 29$			
Reflections collected	42952			
Independent reflections	7954 (R(int) = 0.0678)			
Observed reflections	6443 ($I > 2\sigma(I)$)			
Absorption correction	Semi-empirical from equivalents			
Max. and min. transmission	0.96 and 0.91			
Refinement method	Full-matrix least-squares on F^2			
Data/restraints/parameters	7954 / 1 / 454			
Goodness-of-fit on F^2	0.99			
Final R indices (I>2sigma(I))	R1 = 0.038, wR2 = 0.075			
Absolute structure parameter	0.01(2)			
Largest diff. peak and hole	0.30 and -0.20 $eÅ^{-3}$			

Protein production

Recombinant KLK6 (R74G, R76Q, and N132Q)[7] for biochemical assays was produced as previously described[2] with minor changes. A pFastBac™ donor plasmid including cDNA encoding the sequence for the mature form of KLK6 (with additional R74G, R76Q, and N132Q mutations) preceded at 5′ from the gp64 signal peptide, a 6x His-tag and an enterokinase (EK) recognition sequence of $(Asp)_4Lys$ was purchased from Life Technologies™. This system was transformed in competent *E.coli* DH10MultiBac by electroporation. Transformed cells were grown overnight at 37 °C in 2x TY medium. 5 µL of 1:10 and 1:100 dilution of these cells were cultured on selection plates [YTE Agar + Carbenicillin (100 g/ml), Kanamycin (30 µg/ml), Gentamycin (7 µg/ml), Tetracycline (10 µg/ml), X-gal (400 ug/ml) and IPTG (5 mM)]. White colonies were re-streaked for monoclonal growth on the same plates overnight at 37 °C. Seven white cultures thus obtained were grown in a 3 mL culture for 24 h at 37°C in 2x TY supplemented with the above-mentioned antibiotics. Bacmid DNA containing the KLK6 constructs was isolated using Qiagen's miniprep kit following producer instructions and sequenced. DNA concentrations were determined using NanoDrop spectrophotometer (Thermo Scientific, MA, USA) and the construct was stored at 4 °C prior to use. Sf21 cells were seeded in 6-well plates at 6 x 10^5 cells in 2 mL sf-900 II serum-free media (Gibco, Thermo Fisher) per well and let 15 min for adherence. To a mixture of FuGENE® (Promega) in medium was added each Bacmid construct separately and incubated 15 min at rt. 116 µL of this mixture was added to each well (constructs were added in duplicates) and cells were cultured at 27 °C for 48–60 h. V_0 viral stock obtained from this culture cycle was used to produce high titer V_1 stock from a new culture cycle in Sf21 cells cultured for 2 days after growth arrest (which was also used to test the protein expression on a small scale). The TniHi5 insect cell line was used for production of expressed protein by the viral stock and grown in Express Five™ SFM (Gibco, Thermo Fisher) for 72 h after cell cycle arrest. Cell culture supernatant was collected, filtered and loaded on an ÄKTA™ protein purification system loaded with a prepacked 5 mL cOmplete™ His-Tag Purification Column (Roche) equilibrated with 50 mM Tris/HCl, 250 mM NaCl pH 8.0. Flow-through was discarded and the protein was eluted from the column using 300mM imidazole elution buffer. Fractions containing protein as analyzed by SDS-PAGE were pooled and incubated overnight with 100 µL enterokinase,

dializing in 1 L 50 mM Tris/HCl, 250 mM NaCl pH 8.0 buffer at 4 °C. The obtained solution was again purified with 5 mL cOmplete™ His-Tag Purification Column collecting the flow-through which contains the active KLK6 protein construct deprived of the His-tag. Fractions containing protein as analyzed by SDS-PAGE were pooled and dialyzed overnight with 1 L 50 mM Tris-HCl, 50 mM NaCl pH 7.0 buffer. The protein of interest was further purified by ion-exchange chromatography (5 mL HiTrapQ, 50 mM Tris-HCl, 1 M NaCl pH 7.0 elution buffer) followed by size exclusion chromatography (Superdex 75 10/300 GL, Aldrich®, 50 mM Tris-HCl, 50 mM NaCl pH 7.0 buffer). Final protein purity was determined by SDS-PAGE and ESI-MS, the protein concentration was determined by Bradford assay.

Recombinant KLK6 (R74G, R76Q, and N132Q)[7] for crystallization was produced as described in the following: A triple mutant variant of KLK6 (R74G, R76Q, and N132Q) as cloned in a pXLG (PMID: 18617574) mammalian expression plasmid. The KLK6 expression construct was designed with the native signal peptide of KLK6 (1-16 aa) at the N-terminus, followed by a 6×His tag, tobacco etch virus (TEV) protease cleavage site, a short linker, an enterokinase cleavage site and the KLK6 (22-244 aa). The TEV site allows the removal of the 6×His affinity tag and leads to inactive KLK6 protein. Upon enterokinase treatment, an active KLK6 protein form is obtained. KLK6 protein was transiently expressed using suspension adapted Expi293F™ cells (Thermo Fisher) grown in 600 mL TubeSpin® Bioreactor (TPP) with 200-350 mL FreeStyle ™ 293 expression medium (Thermo Fisher). Cells were kept in a logarithmic phase with cell density between 0.3-2.2×10^6 cells/ml and viability above 98-99%. The transient transfection was performed following the high cell density protocol (PMID: 18617574). Briefly, the cells were spun down for 5 min at 150×g at 22 °C and resuspended to 20×10^6 cells/ml using a fresh medium. Next 20 µg/ml plasmid DNA and 40 µg/ml of PEI (1mg/ml stock solution) (Polyethylenimine, Linear, MW 25000, cat # 23966-1, Polysciences) were added directly to the cells. The cells were incubated for 1 h, before diluting to 1×10^6 cells/ml using pre-warmed medium. To boost the KLK6 expression, 2 mM sodium butyrate was added 12 h post-transfection. Next the protein expression was performed for an additional 60 h at 37 °C in an atmosphere with 8% CO_2 and 70% humidity with orbital shaking at 180 rpm and 25 mm shaking throw (Minitron incubator, INFORS HT). The cells were harvested by centrifuged for 20 mins at 3200xg at 4 °C. Next, the protein-containing medium was mixed with 10×Ni-NTA buffer (500 mM Tris-HCl pH 8 (4 °C), 1.5 M NaCl, 200 mM Imidazole). The medium was applied on a 15 mL Poly-Prep ® Chromatography Columns (Bio-Rad) containing 0.5-1.0 mL settled Ni-NTA agarose beads (Qiagen, #30210), pre-equilibrated with wash buffer (50 mM Tris-HCl pH 8 (4 °C), 150 mM NaCl, 20 mM Imidazole). Unbound protein was removed with 20 column volumes (CV) of wash buffer (50 mM Tris-HCl pH 8 (4 °C), 150 mM NaCl, 20 mM Imidazole) followed by elution of KLK6 protein by incubation for 5 mins with 6 CV elution buffer (50 mM Tris-HCl pH 8 (4 °C), 150 mM NaCl, 250 mM Imidazole) and collecting the eluted protein. Fractions containing KLK6 protein were pooled together and supplemented with 2 mM $CalCl_2$ and 50 µl (800 U) of Enterokinase protease (NEB, # P8070L). The sample was dialyzed against 1 L dialysis buffer (20 mM Tris-HCl pH 8 (4 °C), 150 mM NaCl, 20 mM Imidazole, 2 mM $CaCl_2$) at 8 °C for 16 h with gently mixing. Uncleaved KLK6 protein was removed by applying the sample on the Ni-NTA column. Next, the protein sample was concentrated with Amicon Ulta centrifugal Filters with 10K cut-off (Merck Millipore) and further purified by size exclusion chromatography using Superdex 75 10/300 GL (Cytiva) and buffer containing 50 mM Tris-HCl pH 7.5 (22 °C), 50 mM NaCl buffer. Fractions containing KLK6 were pulled together and concentrated up to 8.4 - 11.00 mg/ml as described above. Next, the protein was flash-frozen in a thin wall, low protein binding 0.2 ml tubes using liquid nitrogen and stored at -80°C until further use.

Protein crystallography

KLK in complex with the ligands was crystallized in previously published condition with 0.1 M Tris-HCl pH 8.5, 0.20 M trimethylamine N-oxide and 18-24% PEG MME 2000 but in complex with DKFZ-878 (structure see below) instead of benzamidine.[8] Before crystallization, KLK6 at 10 mg/ml was

incubated with 5 mM **DKFZ-878**. Crystals were obtained by sitting drop vapour diffusion with 200 nL of protein and 200 nL mothor liquor and the crystals appeared in 3-4 days at 20 °C. Crystals were then soaked in 1mM of **DKFZ-917** or **DKFZ-918** for 2-3 days. The crystals were cryoprotected in mother liquor supplemented with 30% glycerol and the inhibitor and snap frozen in liquid nitrogen. Several datasets were collected at the ID23-2 beamline at European synchrotron research facility (ESRF), Grenoble, France. Datasets were indexed and scaled with XDS[9] and the structures were solved by molecular replacement with the previously published crystal structure of KLK6 (PDB ID: 3VFE)[8] as the search model in Phaser.[10] Model building was performed in coot[11] and structures were refined using phenix.refine from the Phenix suite.[12]

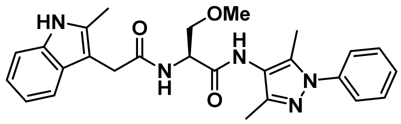

DKFZ-878

Data collection and refinement statistics.

	KLK6+**DKFZ-918**	KLK6+**DKFZ-917**
Wavelength	0.87 Å	0.87 Å
Resolution range	27.57 - 1.88 (1.948 - 1.88) Å	36.03 - 1.5 (1.554 - 1.5) Å
Space group	P 1 21 1	P 21 21 21
Unit cell	62.41 46.86 68.23 90 92.319 90	44.5 48.23 108.39 90 90 90
Total reflections	208034 (15989)	183074 (8351)
Unique reflections	32014 (2942)	37067 (3140)
Multiplicity	6.5 (5.4)	4.9 (2.7)
Completeness (%)	99.01 (92.14)	97.06 (84.07)
Mean I/sigma(I)	9.31 (0.90)	8.74 (0.85)
Wilson B-factor	35.33	15.57
R-merge	0.11 (1.21)	0.22 (0.96)
R-meas	0.12 (1.33)	0.24 (1.16)
R-pim	0.05 (0.557)	0.10 (0.65)
CC1/2	0.998 (0.51)	0.988 (0.35)
CC*	0.999 (0.82)	0.997 (0.72)
Reflections used in refinement	32007 (2942)	37059 (3140)
Reflections used for R-free	1599 (147)	1854 (157)
R-work	0.209 (0.328)	0.182 (0.314)
R-free	0.241 (0.335)	0.218 (0.348)

Number of non-hydrogen atoms	3535	2032
macromolecules	3315	1713
ligands	62	37
solvent	158	282
Protein residues	439	222
RMS(bonds)	0.027	0.018
RMS(angles)	2.78	1.82
Ramachandran favored (%)	96.30	98.64
Ramachandran allowed (%)	3.46	1.36
Ramachandran outliers (%)	0.23	0.00
Rotamer outliers (%)	1.96	0.00
Average B-factor	51.36	20.93
macromolecules	51.68	19.24

Biochemical Assay

KLK6 enzymatic activity was monitored in vitro by cleavage of the fluorogenic substrate N-Boc-FSR-AMC (Bachem) with a monochromator CLARIOstar® platereader (BMG LABTECH). To each well of a 384-well black plate containing 20 µL KLK6 (final assay concentration 10 nM) in reaction buffer (50 mM Tris, 150 mM NaCl, 1 mM EDTA, 0.05% Tween-20, pH 7.5) was added fluorogenic substrate (300 µM) dosed from a 100 mM DMSO stock solution with a D300e Digital Dispenser (Tecan). Immediately afterwards, inhibitors were added at eleven serial dilutions in triplicates ranging from 50 nM – 0.65 nM dosed from 5 µM DMSO stock solutions with a D300e Digital Dispenser (Tecan). Before each Substrate/Inhibitor addition the Digital Dispenser normalized the DMSO concentration to the well with the highest DMSO content (final DMSO volume 1.3%). The plate was shaken (500 rpm orbital shaker, 15 s) before starting a kinetic measurement of fluorescence intensity (ex: 350-15, em: 440-20; every 71 s for 70 min). The slope of the measured signals was calculated from cycles 29 to 59 using GraphPad Prism, normalized over the DMSO control and then used to generate dose-response curves (from two experiments).

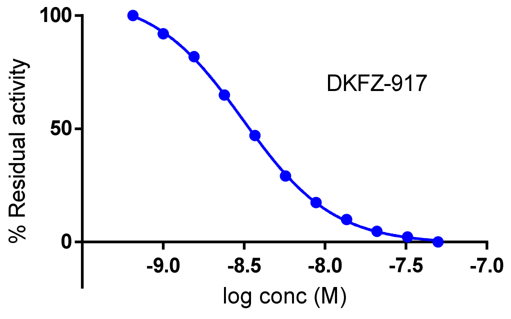

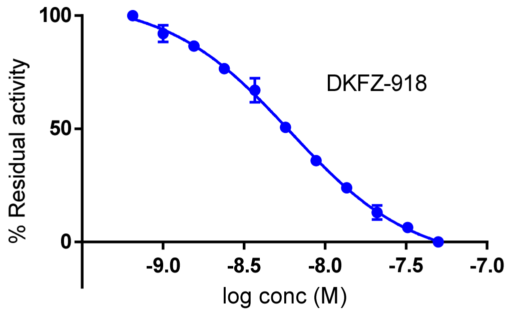

DKFZ-917 pIC_{50} 8.51 (95%CI 8.49 to 8.52)

DKFZ-918 pIC_{50} 8.22 (95%CI 8.18 to 8.25)

Literature

[1] Mukade, T.; Dragoli, D. R.; Ellman, J. A. *J. Comb. Chem.* **2003**, *5*, 590–596.

[2] Liu, L.; Cogan, D. A.; Owens, T. D.; Tang, T. P.; Ellman, J. A. *J. Org. Chem.* **1999**, *64*, 1278–1284.

[3] Hernando, E.; Castillo, R.R.; Rodríguez, N.; Gómez Arrayás, R.; Carretero, J.C. *Chem. Eur. J.* **2014**, *20*, 13854–13859.

[4] L. Krause, R. Herbst-Irmer, G.M. Sheldrick, D. Stalke *J. Appl. Cryst.* **2015**, *48*, 3–10.

[5] G.M. Sheldrick *Acta. Cryst.* **2015**, *A71*, 3–8.

[6] G.M. Sheldrick *Acta. Cryst.* **2015**, *C71*, 3–8.

[7] Liang, G.; Chen, X.; Aldous, S.; Pu, S. F.; Mehdi, S.; Powers, E.; Xia, T.;Wang, R. *Bioorg. Med. Chem. Lett.* **2012**, *22*, 2450–2455.

[8] Bernett, M. J.; Blaber, S. I.; Scarisbrick, I. A.; Dhanarajan, P.; Thompson, S.M.; Blaber, M. *J. Biol. Chem.* **2002**, *277*, 24562–24570.

[9] Kabsch, W. Xds. *Acta Cryst.***2010**, *D66*, 125–132.

[10] McCoy, A. J.; Grosse-Kunstleve R. W.; Adams P.D.; Winn M. D.; Storoni L. C.; Read R. J. *J. Appl. Cryst.* **2007**, *40*, 658–674.

[11] Emsley, P.; Cowtan, K. *Acta Cryst.* **2004**, *D60*, 2126–2132.

[12] Adams, P. D.; Afonine, P. V.; Bunkóczi, G.; Chen, V. B.; Davis, I. W.; Echols, N.; Headd, J. J.; Hung, L. W.; Kapral, G. J.; Grosse-Kunstleve, R. W.; McCoy, A. J.; Moriarty, N. W.; Oeffner, R.; Read, R. J.; Richardson, D. C.; Richardson, J. S.; Terwilliger, T. C.; Zwart, P. H. *Acta Cryst.* **2010**, *D66*, 213–221.

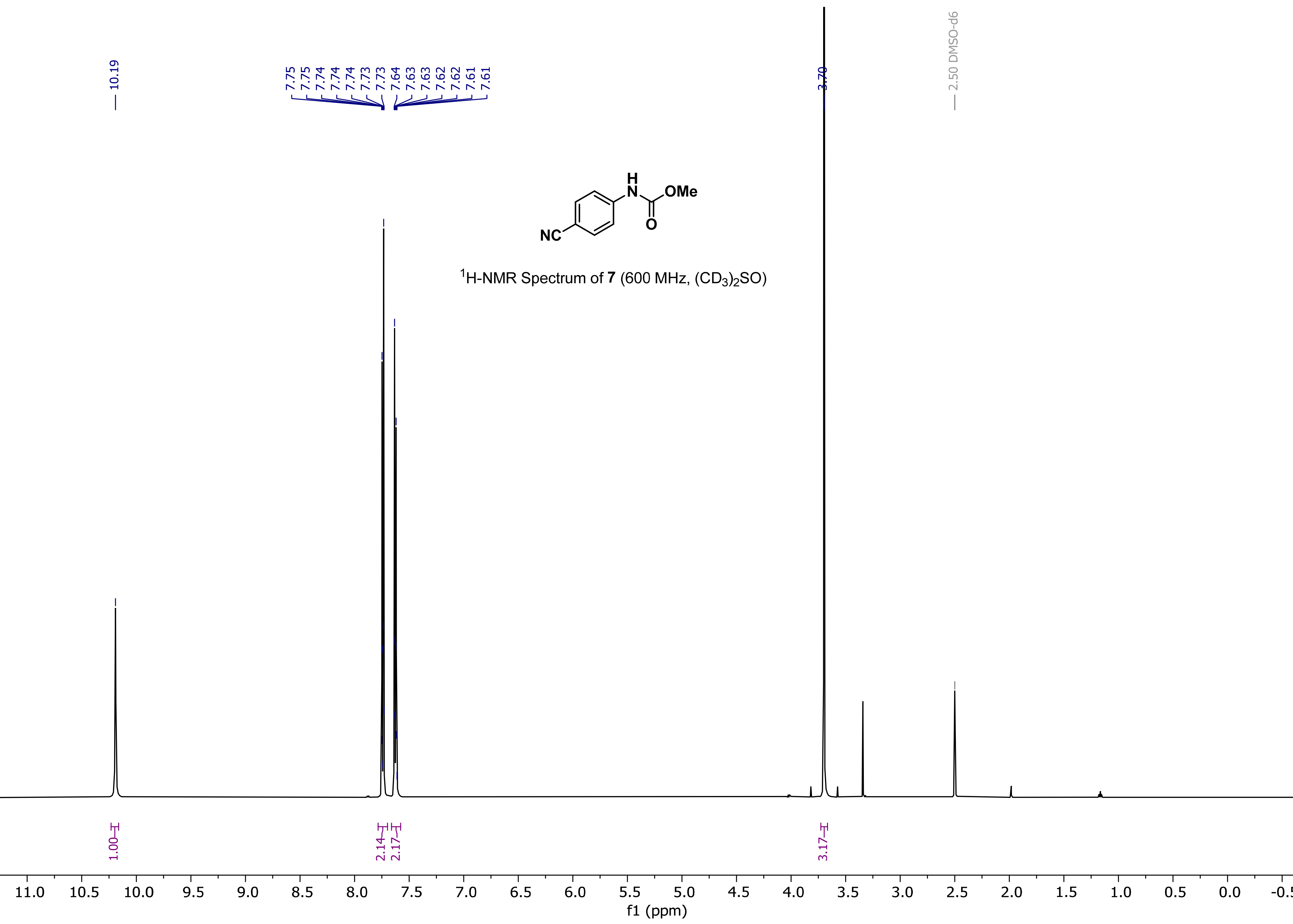

10.19
7.75
7.75
7.74
7.74
7.74
7.73
7.73
7.64
7.63
7.63
7.62
7.62
7.61
7.61
3.70
2.50 DMSO-d6
NC
H
N
OMe
O
1H-NMR Spectrum of 7 (600 MHz, (CD3)2SO)
1.00
2.14
2.17
3.17
11.0
10.5
10.0
9.5
9.0
8.5
8.0
7.5
7.0
6.5
6.0
5.5
5.0
4.5
4.0
3.5
3.0
2.5
2.0
1.5
1.0
0.5
0.0
f1 (ppm)

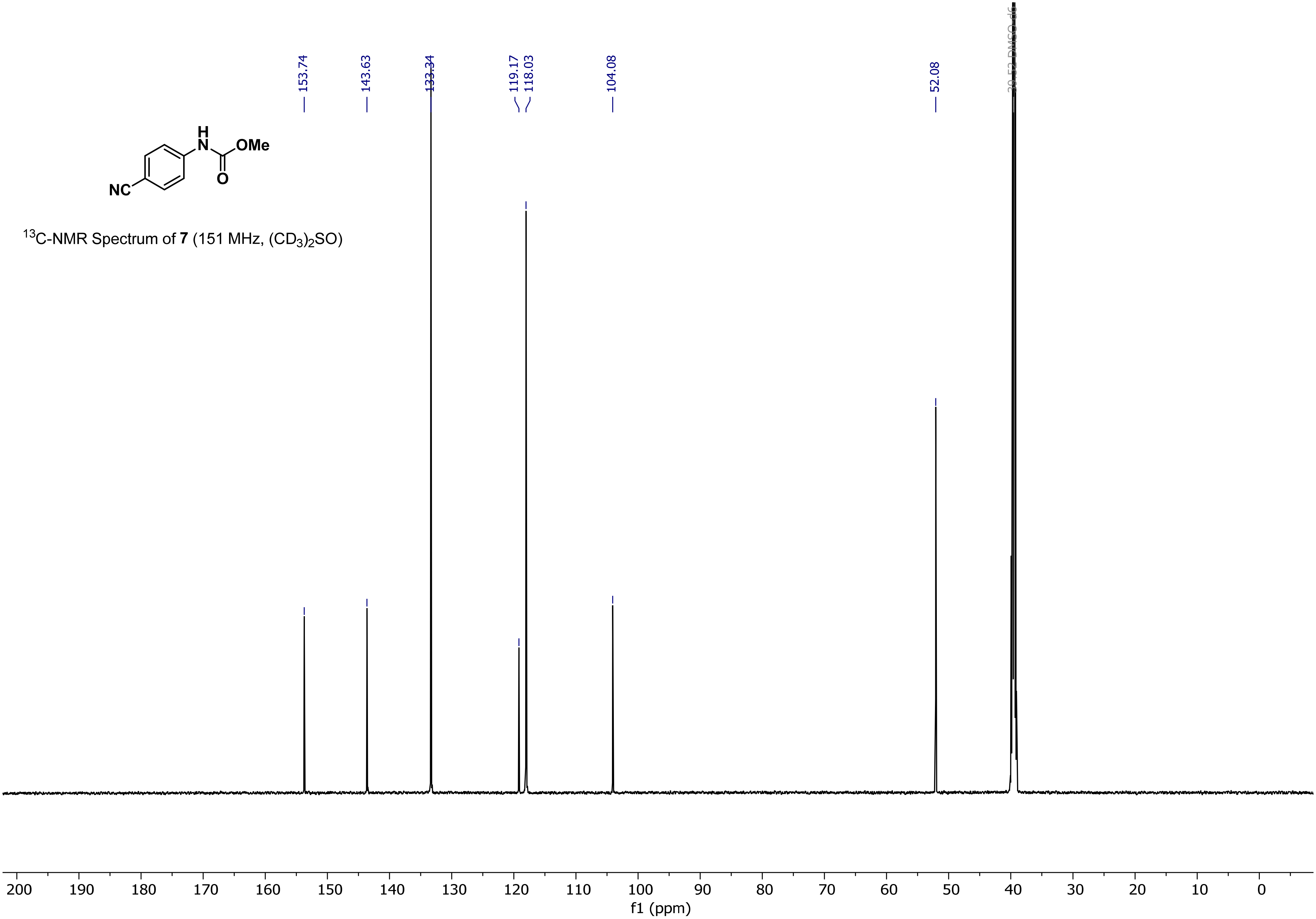

^{13}C-NMR Spectrum of **7** (151 MHz, $(CD_3)_2SO$)

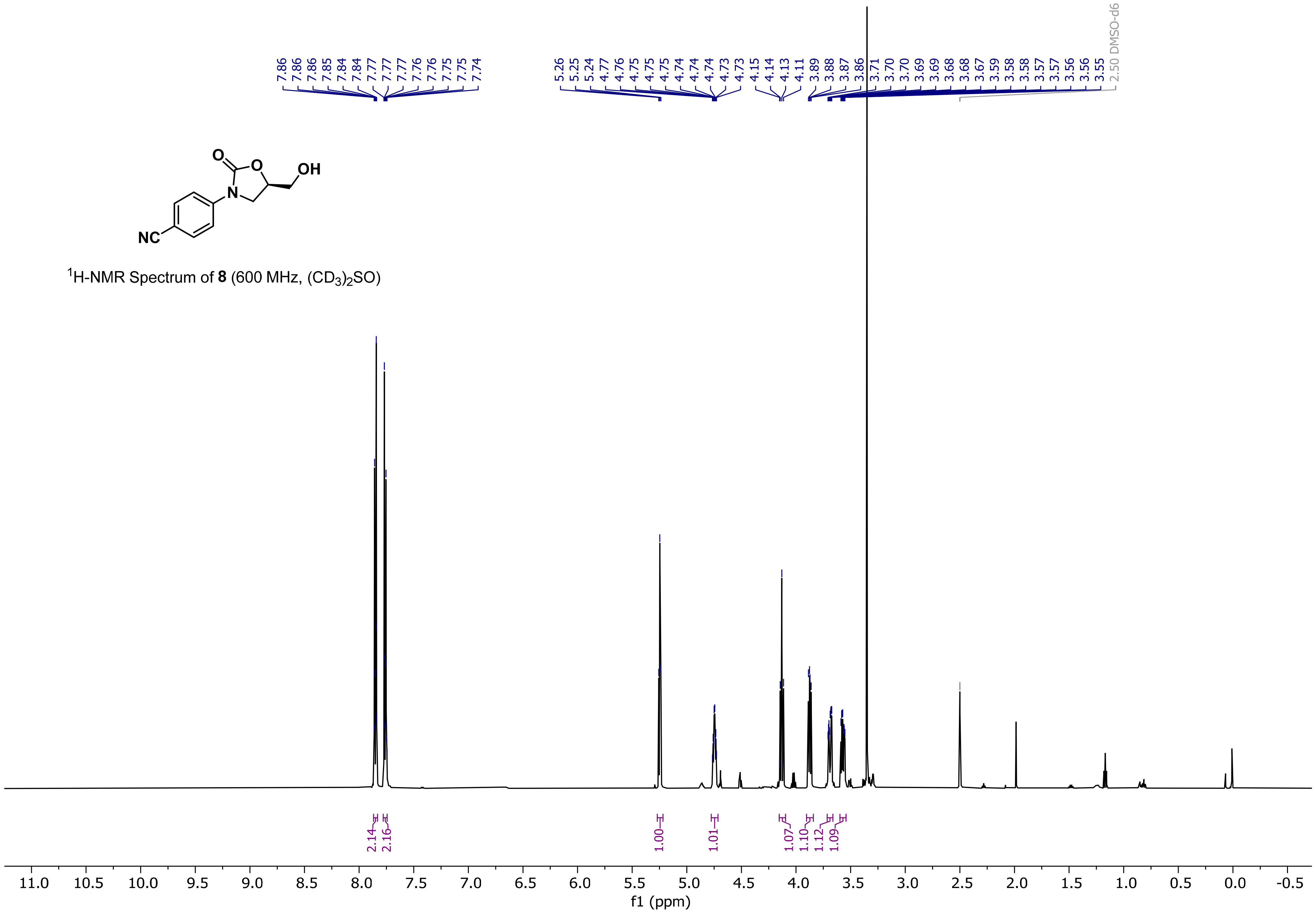

^{1}H-NMR Spectrum of **8** (600 MHz, $(CD_3)_2SO$)

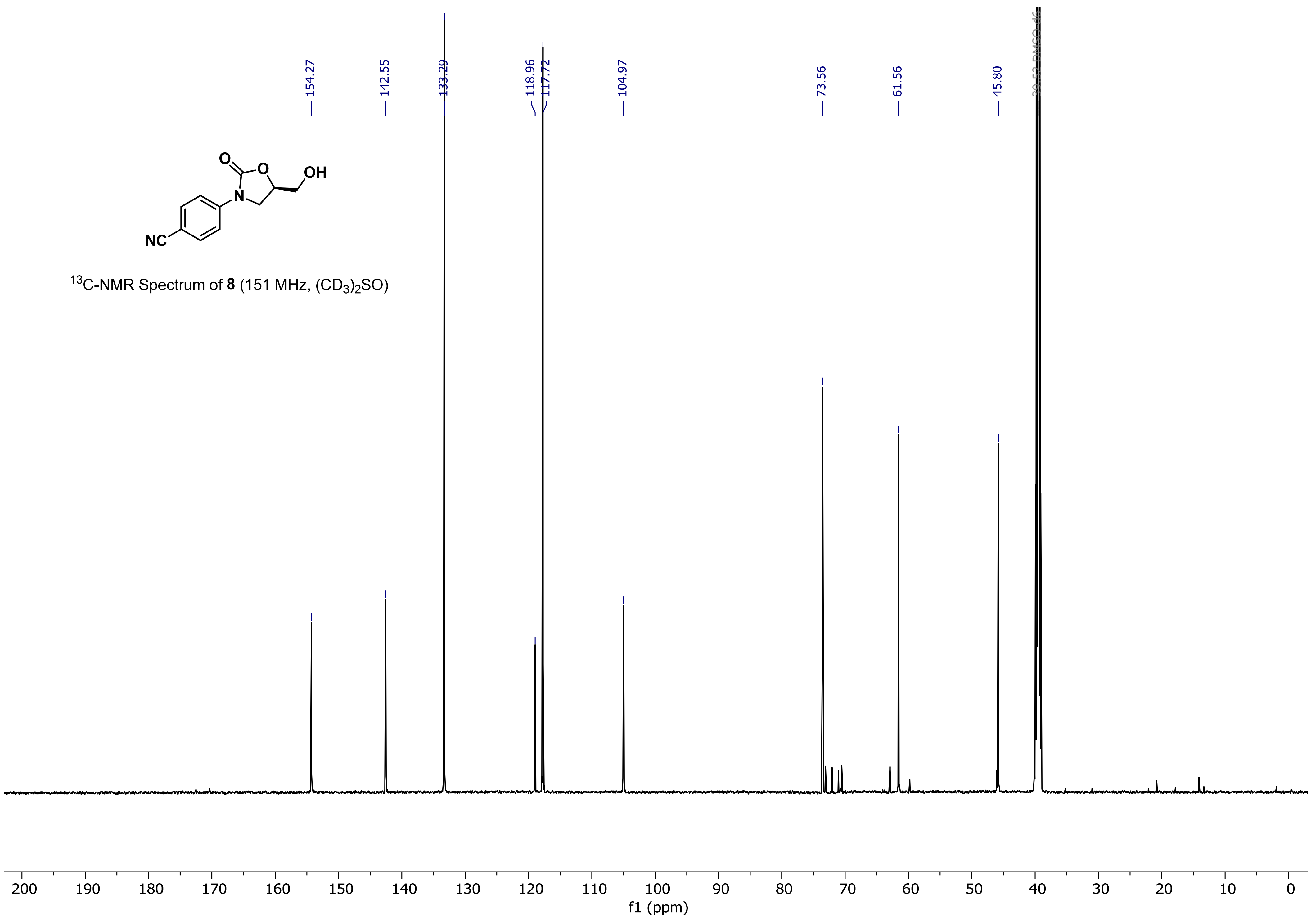

^{13}C-NMR Spectrum of **8** (151 MHz, $(CD_3)_2SO$)

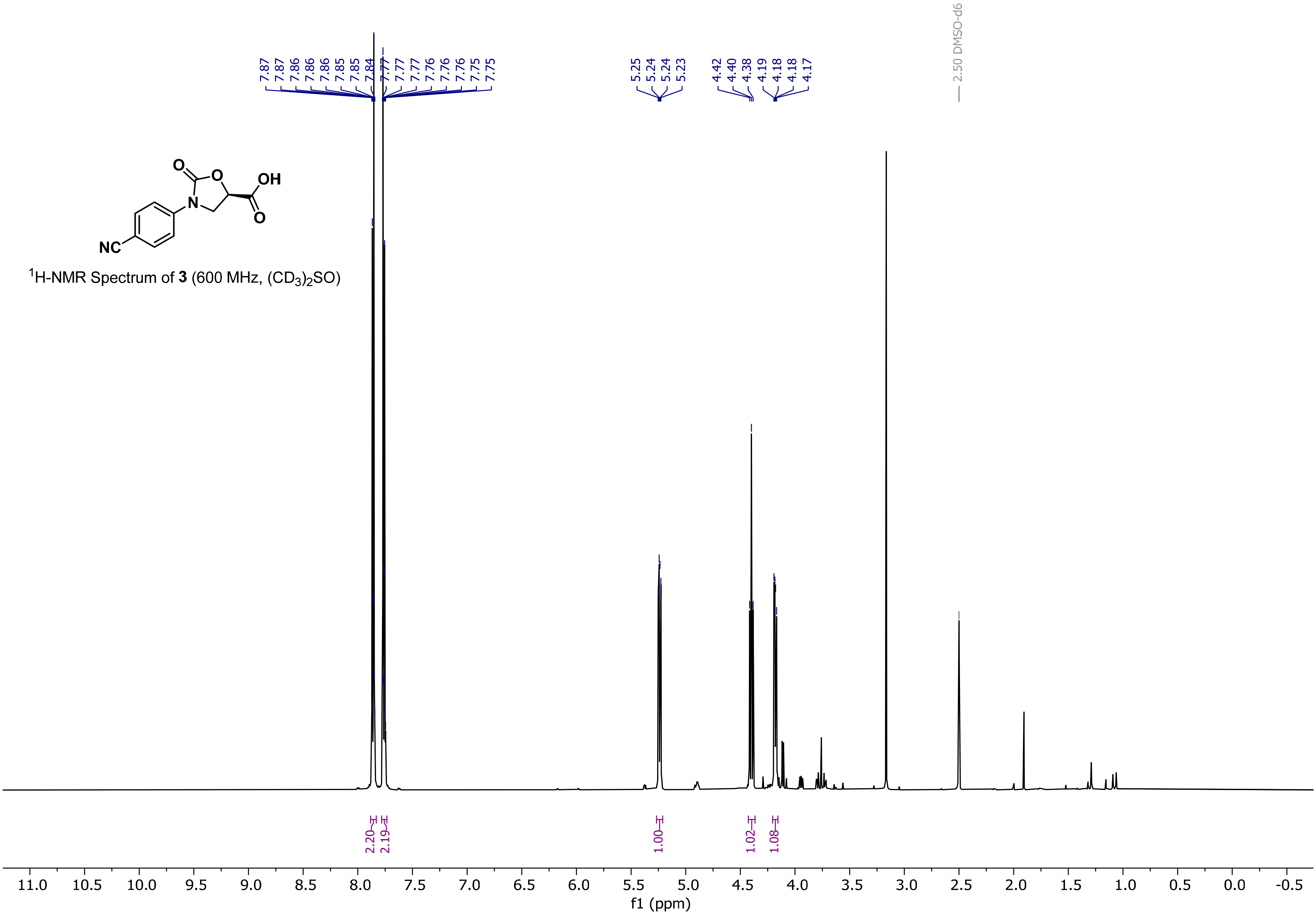

^{1}H-NMR Spectrum of **3** (600 MHz, $(CD_3)_2SO$)

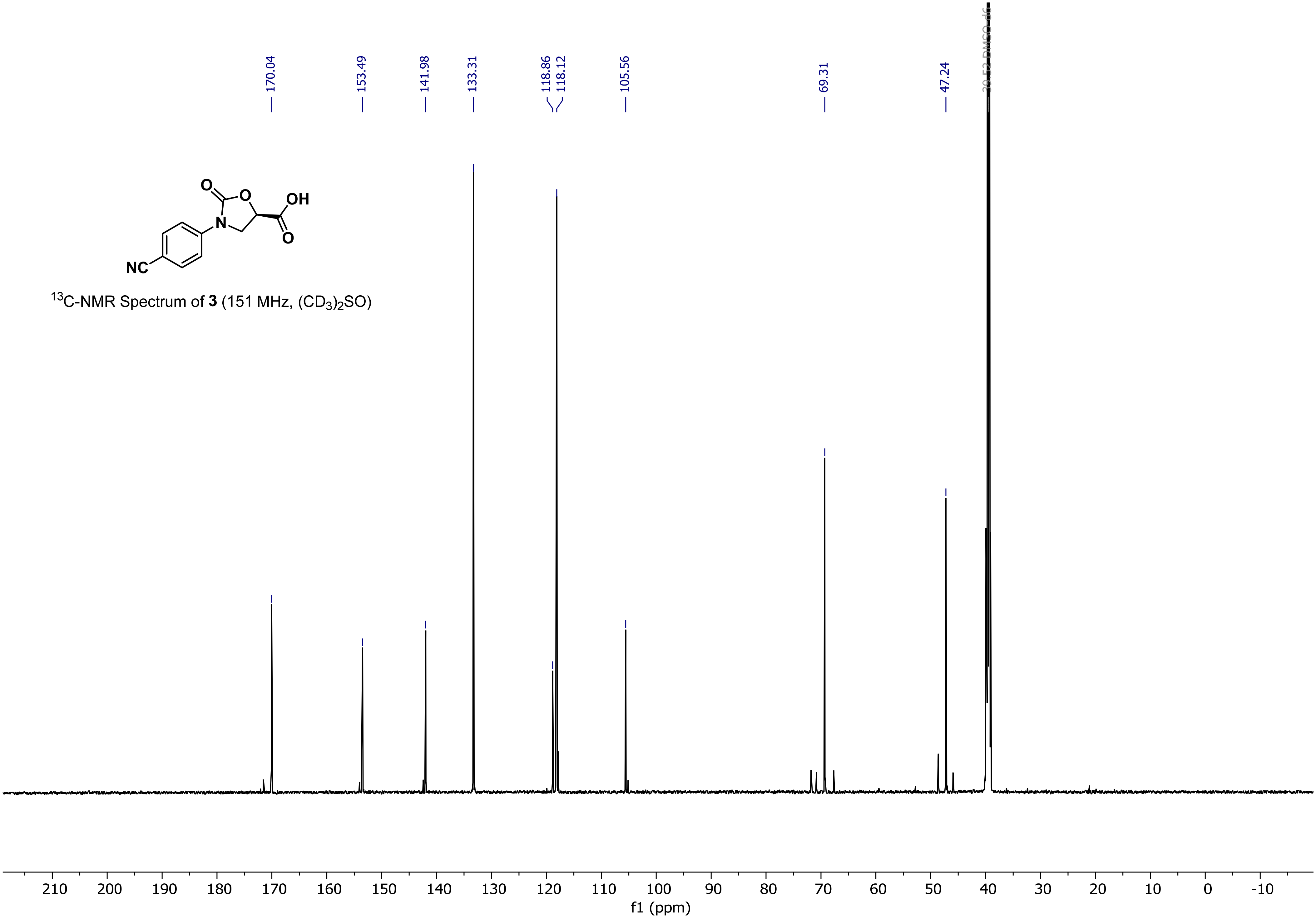

^{13}C-NMR Spectrum of **3** (151 MHz, $(CD_3)_2SO$)

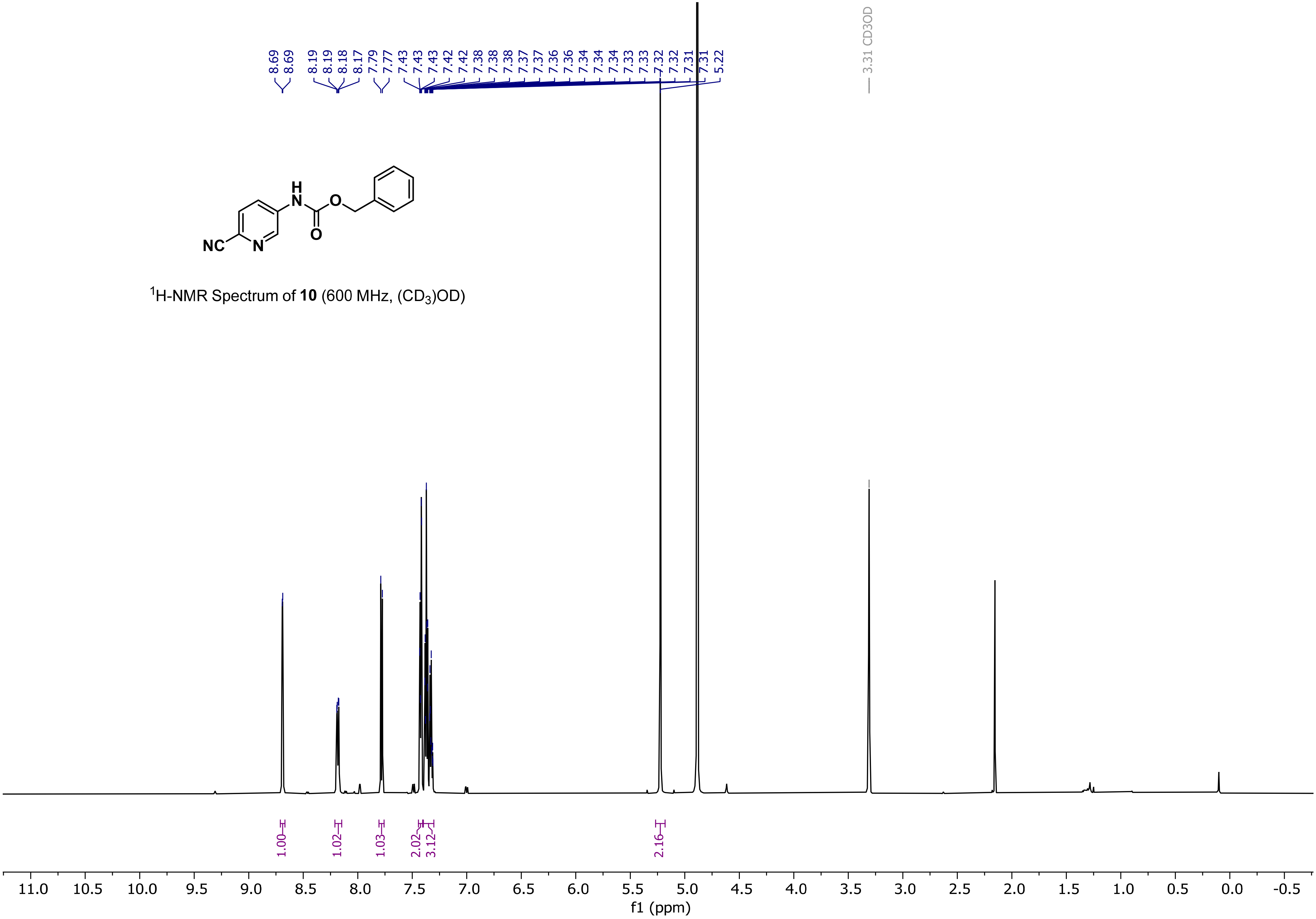

^{1}H-NMR Spectrum of **10** (600 MHz, $(CD_3)OD$)

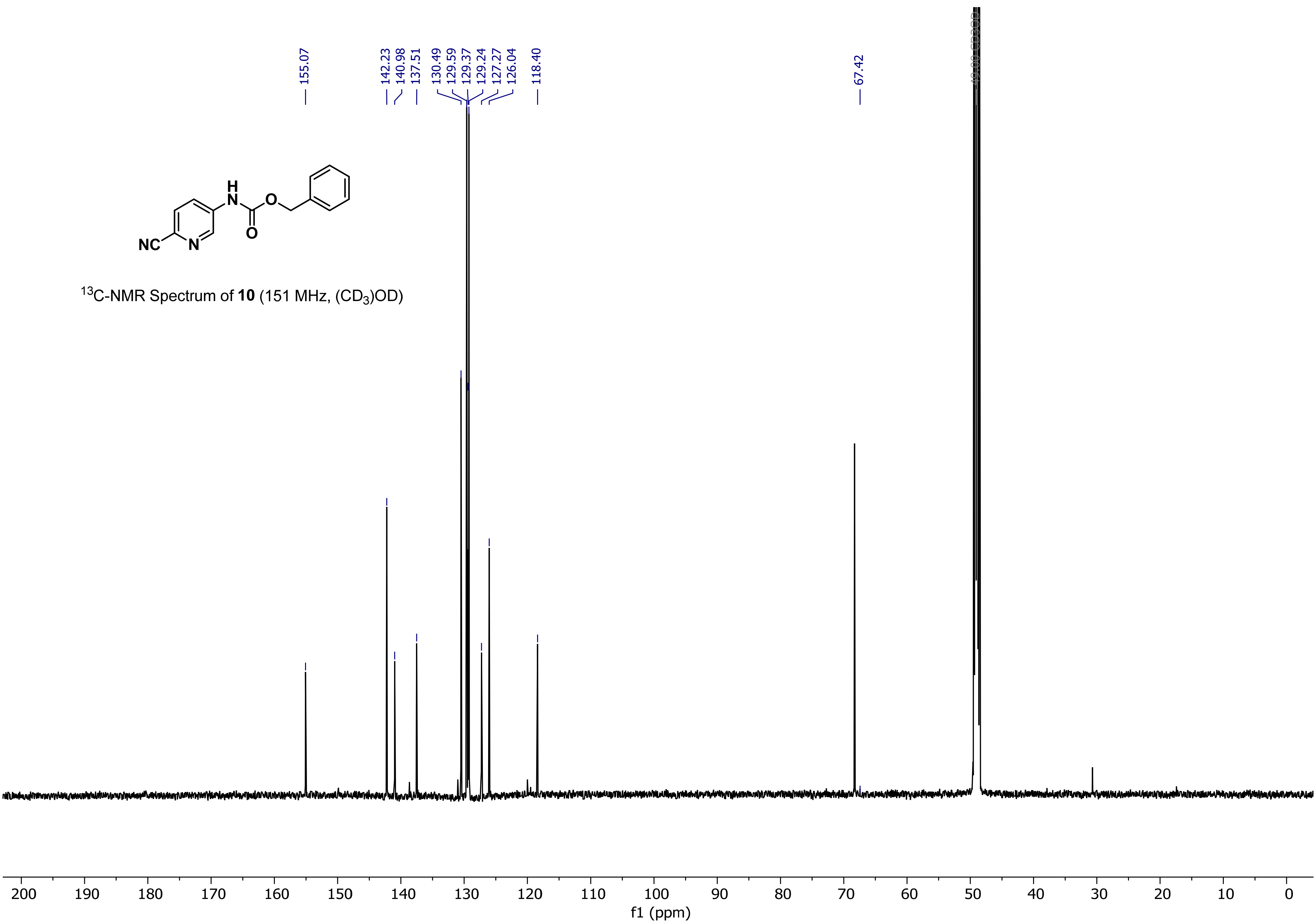

^{13}C-NMR Spectrum of **10** (151 MHz, $(CD_3)OD$)

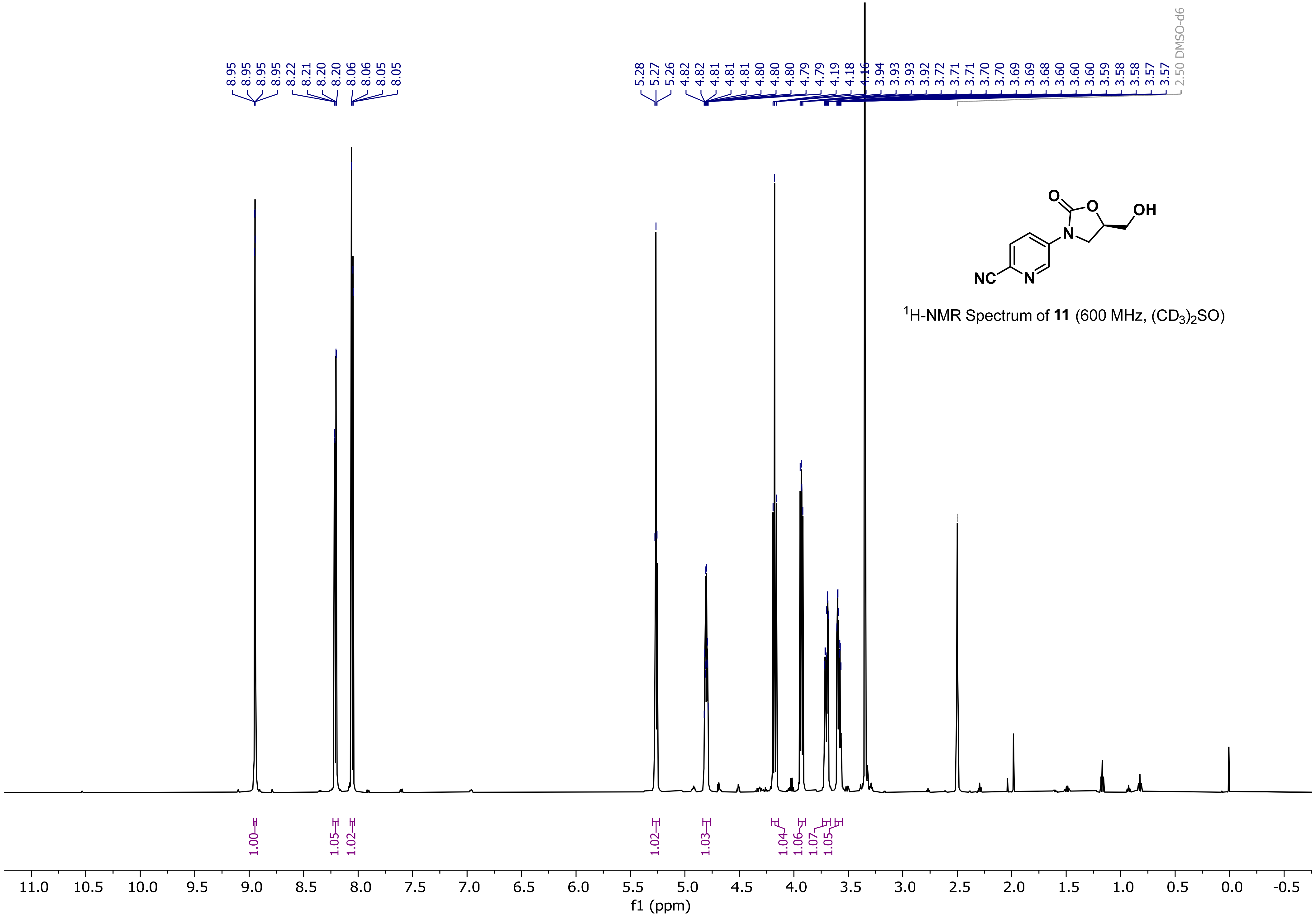

^{1}H-NMR Spectrum of **11** (600 MHz, $(CD_3)_2SO$)

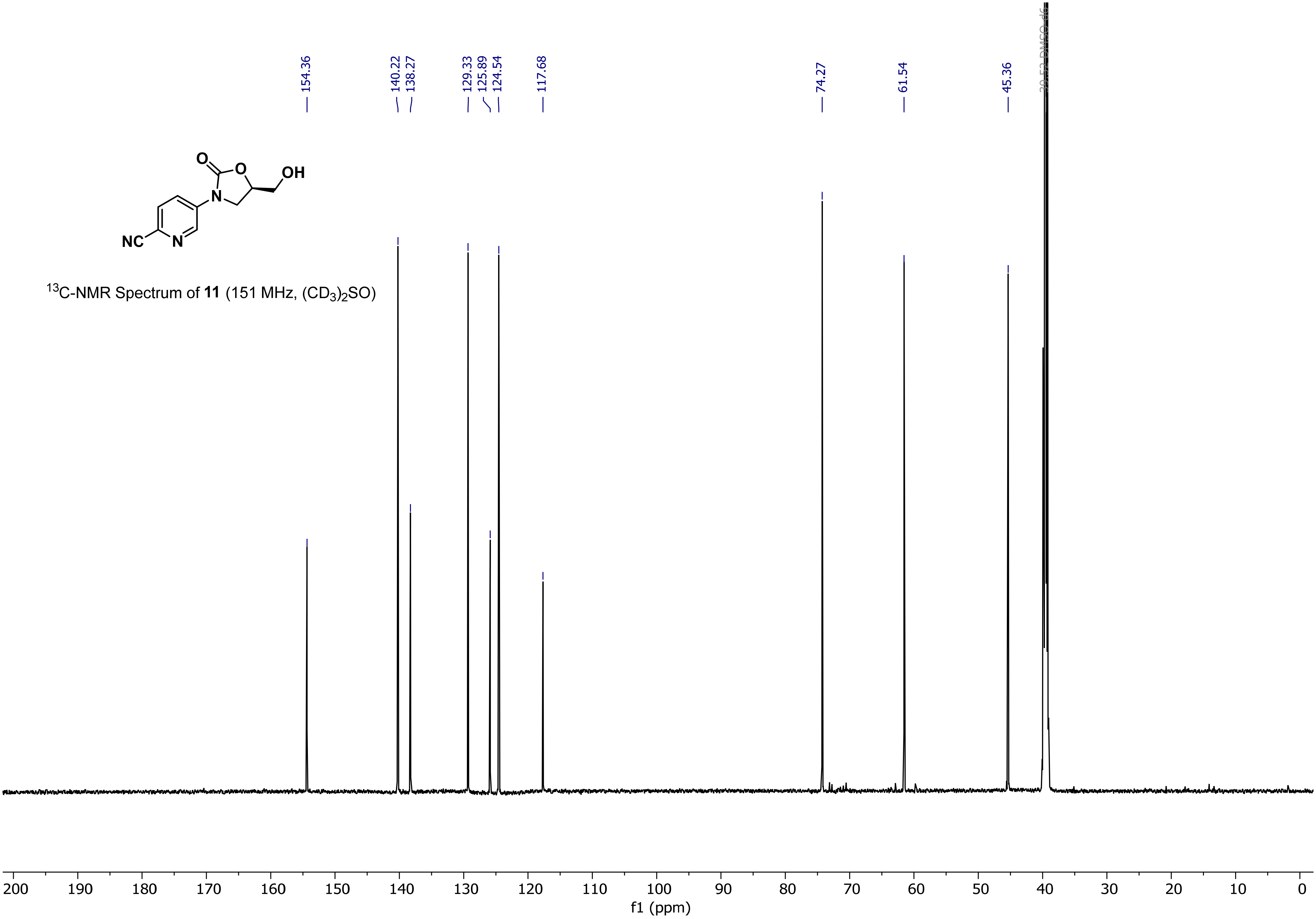

154.36
140.22
138.27
129.33
125.89
124.54
117.68
74.27
61.54
45.36
O
O
OH
N
NC
N
^{13}C-NMR Spectrum of **11** (151 MHz, $(CD_3)_2SO$)
200
190
180
170
160
150
140
130
120
110
100
90
80
70
60
50
40
30
20
10
0
f1 (ppm)

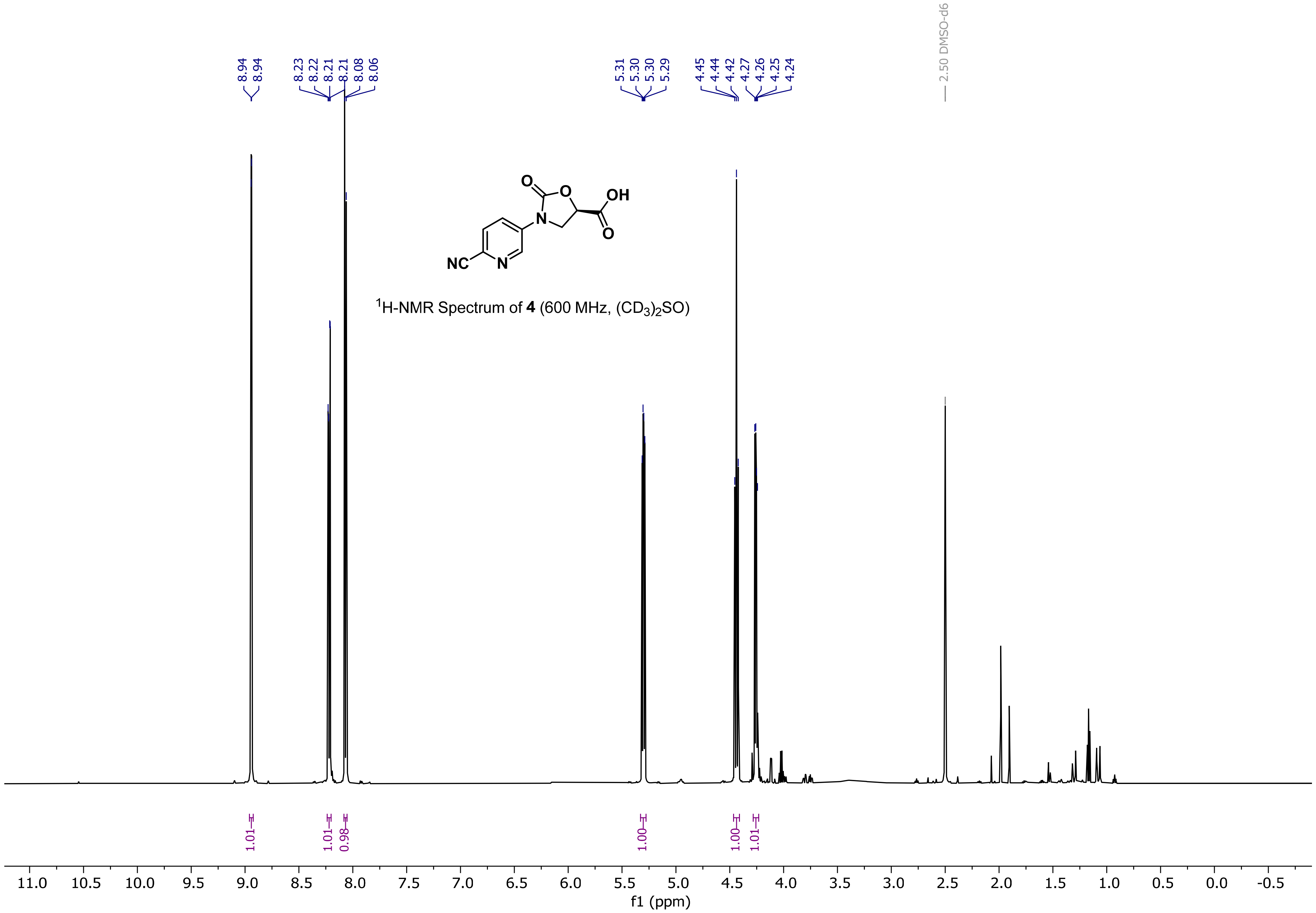

^{1}H-NMR Spectrum of **4** (600 MHz, $(CD_3)_2SO$)

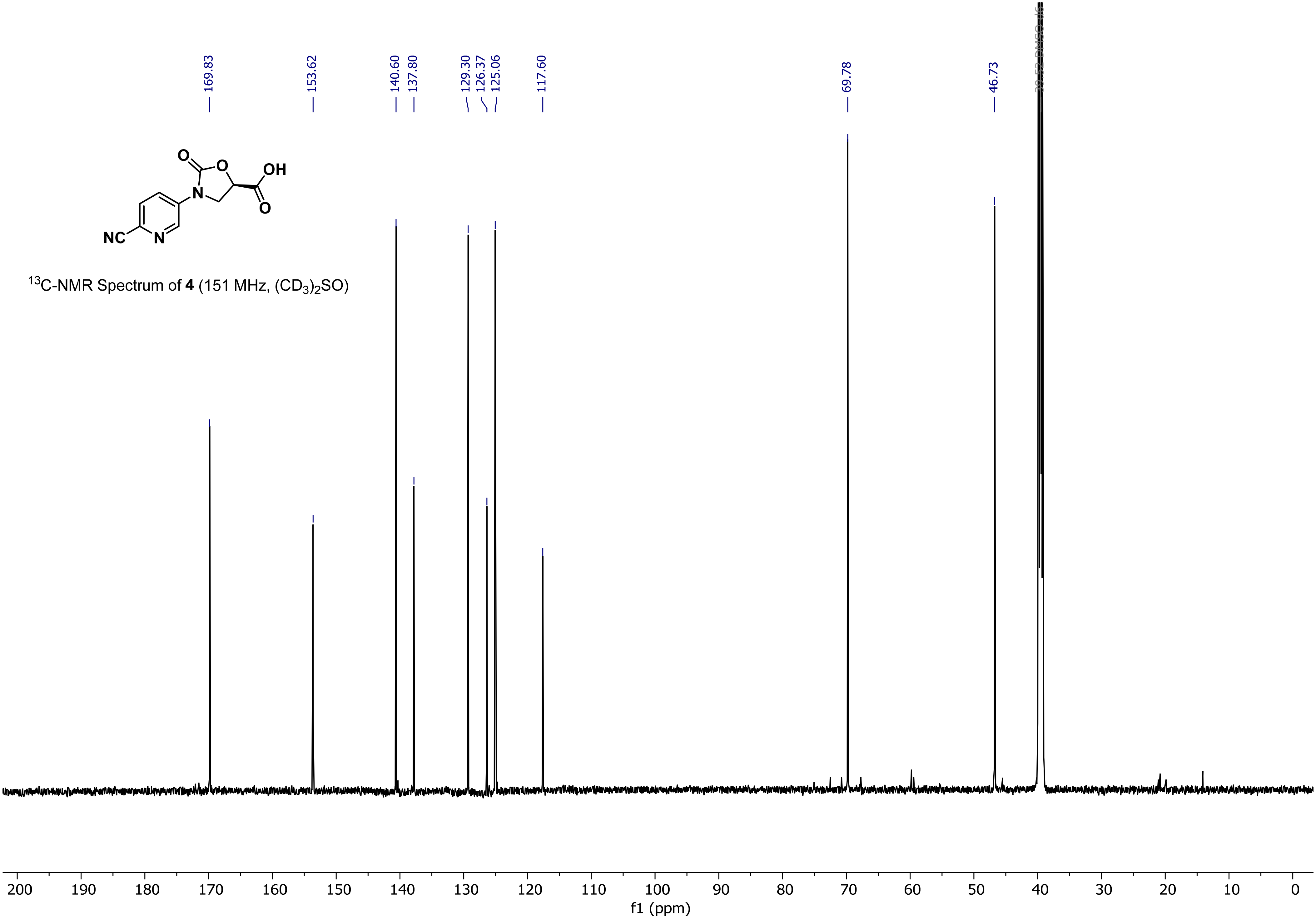

^{13}C-NMR Spectrum of **4** (151 MHz, $(CD_3)_2SO$)

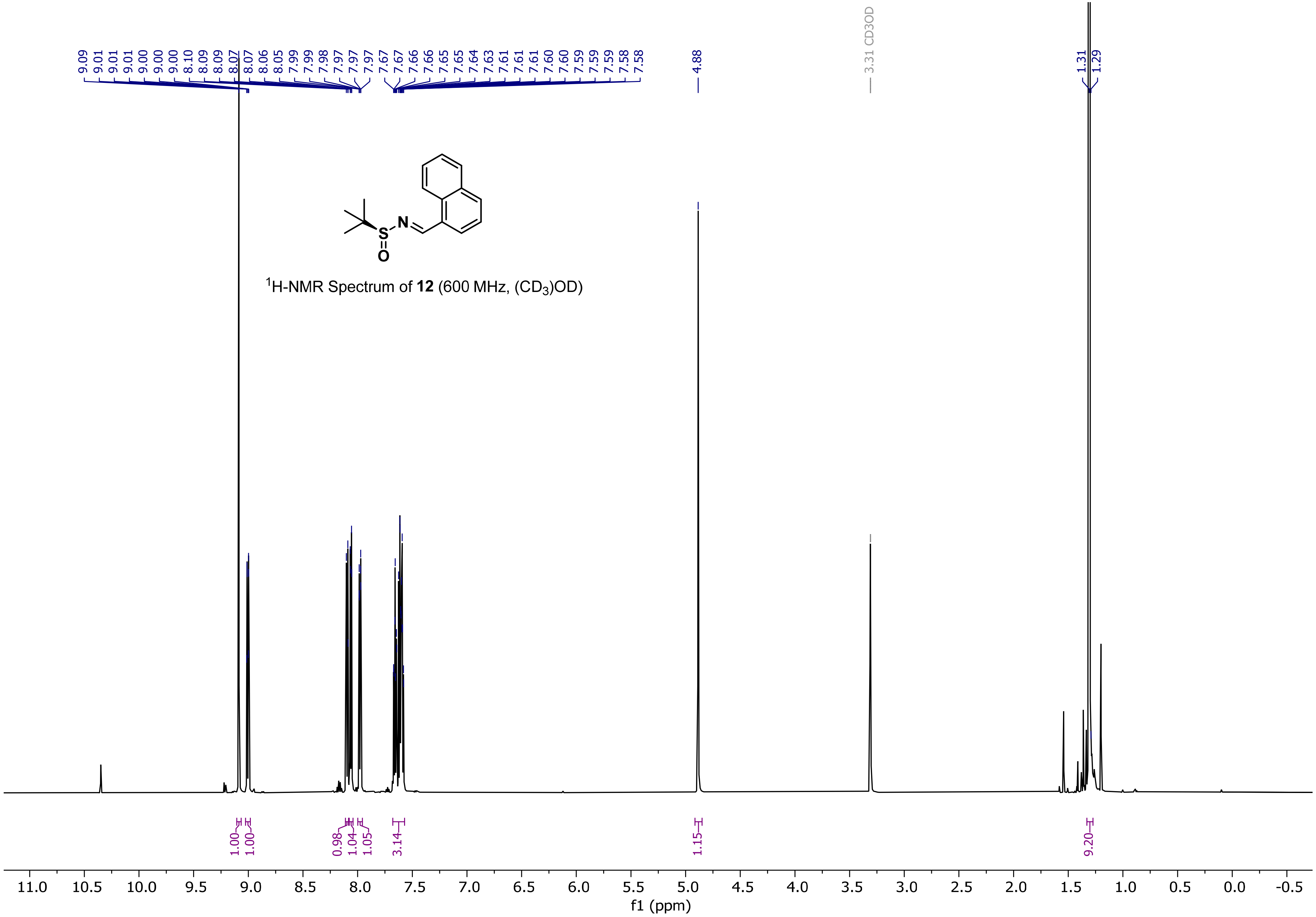

^{1}H-NMR Spectrum of **12** (600 MHz, $(CD_3)OD$)

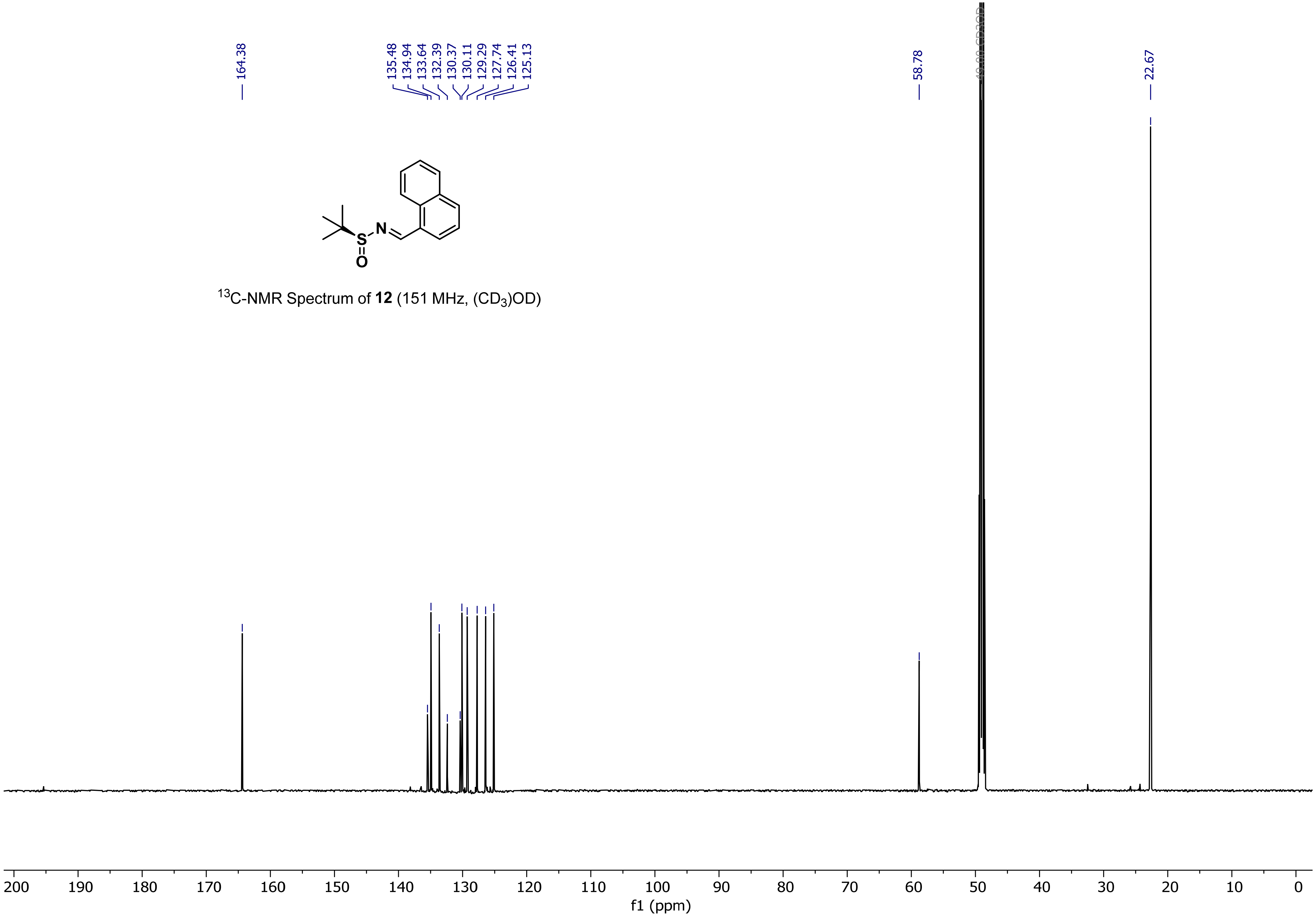

^{13}C-NMR Spectrum of **12** (151 MHz, $(CD_3)OD$)

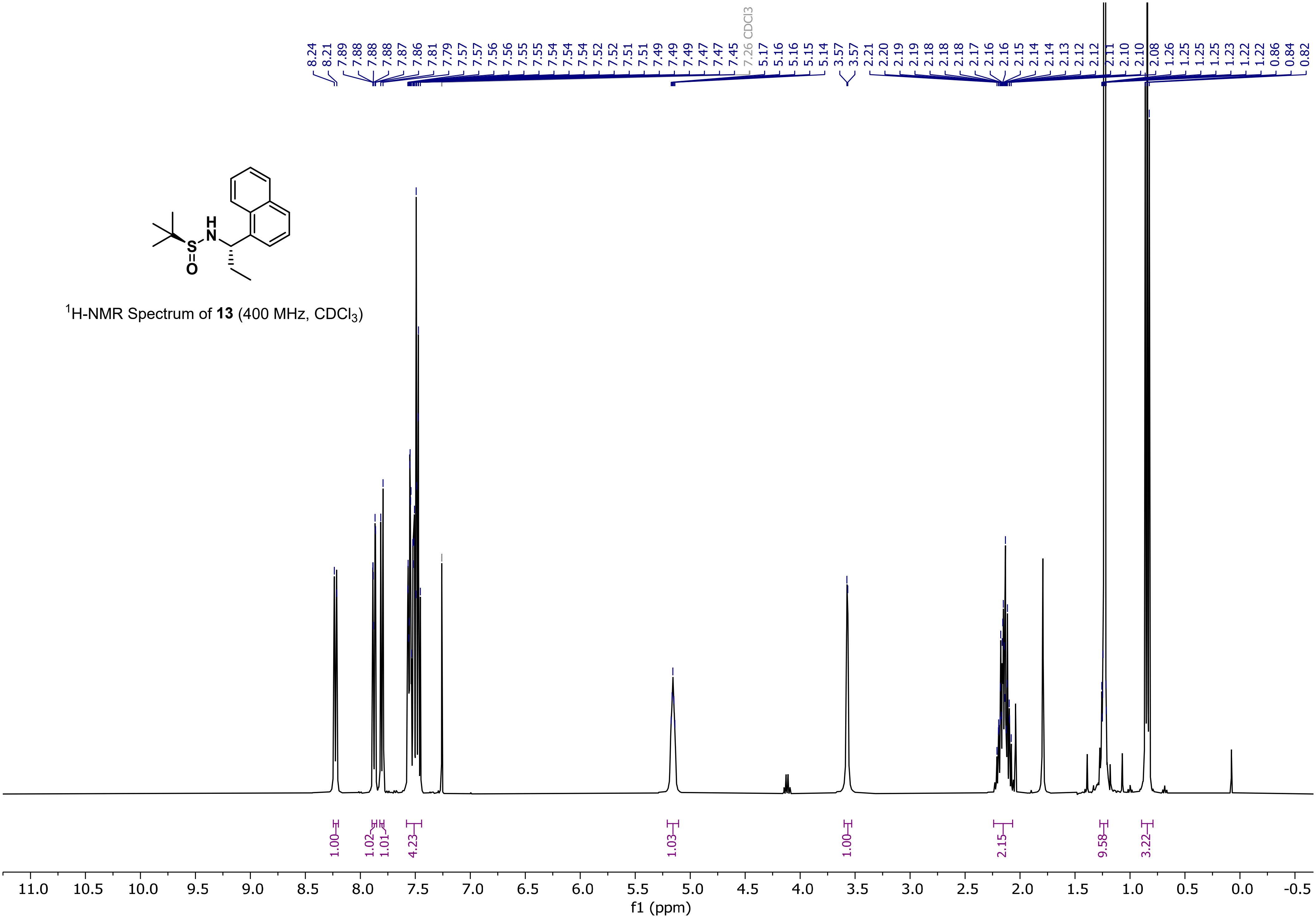

8.24 8.21 7.89 7.88 7.88 7.88 7.87 7.86 7.81 7.79 7.57 7.57 7.56 7.56 7.55 7.55 7.54 7.54 7.54 7.52 7.52 7.51 7.51 7.49 7.49 7.47 7.47 7.45 7.26 CDCl3 5.17 5.16 5.16 5.15 5.14 3.57 3.57 2.21 2.20 2.19 2.19 2.18 2.18 2.18 2.17 2.16 2.16 2.15 2.14 2.14 2.13 2.12 2.12 2.11 2.10 2.10 2.08 1.26 1.25 1.25 1.25 1.23 1.22 1.22 0.86 0.84 0.82
H
N
S
O
^{1}H-NMR Spectrum of **13** (400 MHz, $CDCl_3$)
1.00 1.02 1.01 4.23 1.03 1.00 2.15 9.58 3.22
11.0 10.5 10.0 9.5 9.0 8.5 8.0 7.5 7.0 6.5 6.0 5.5 5.0 4.5 4.0 3.5 3.0 2.5 2.0 1.5 1.0 0.5 0.0 -0.5
f1 (ppm)

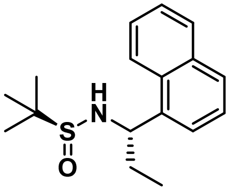

^{13}C-NMR Spectrum of **13** (101 MHz, $CDCl_3$)

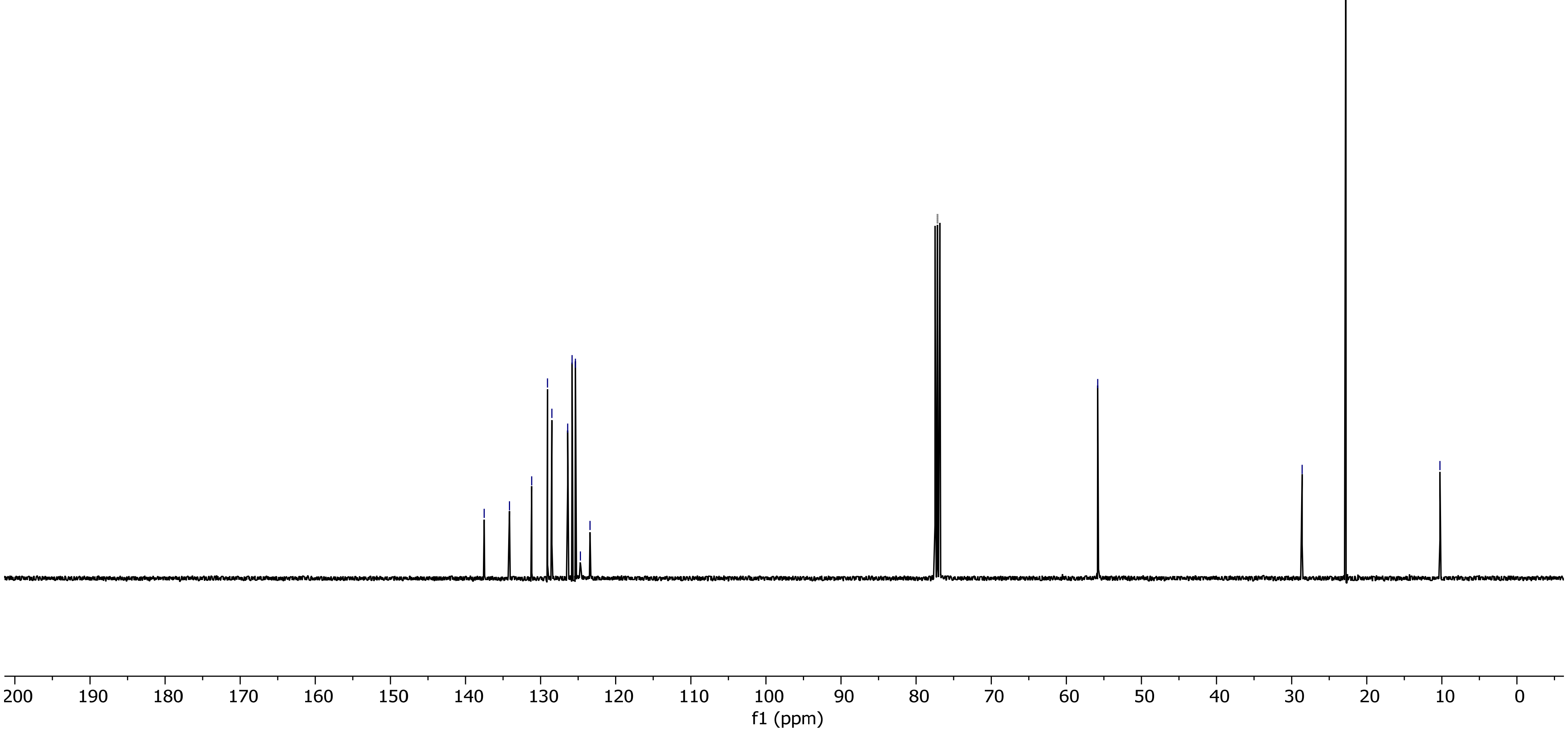

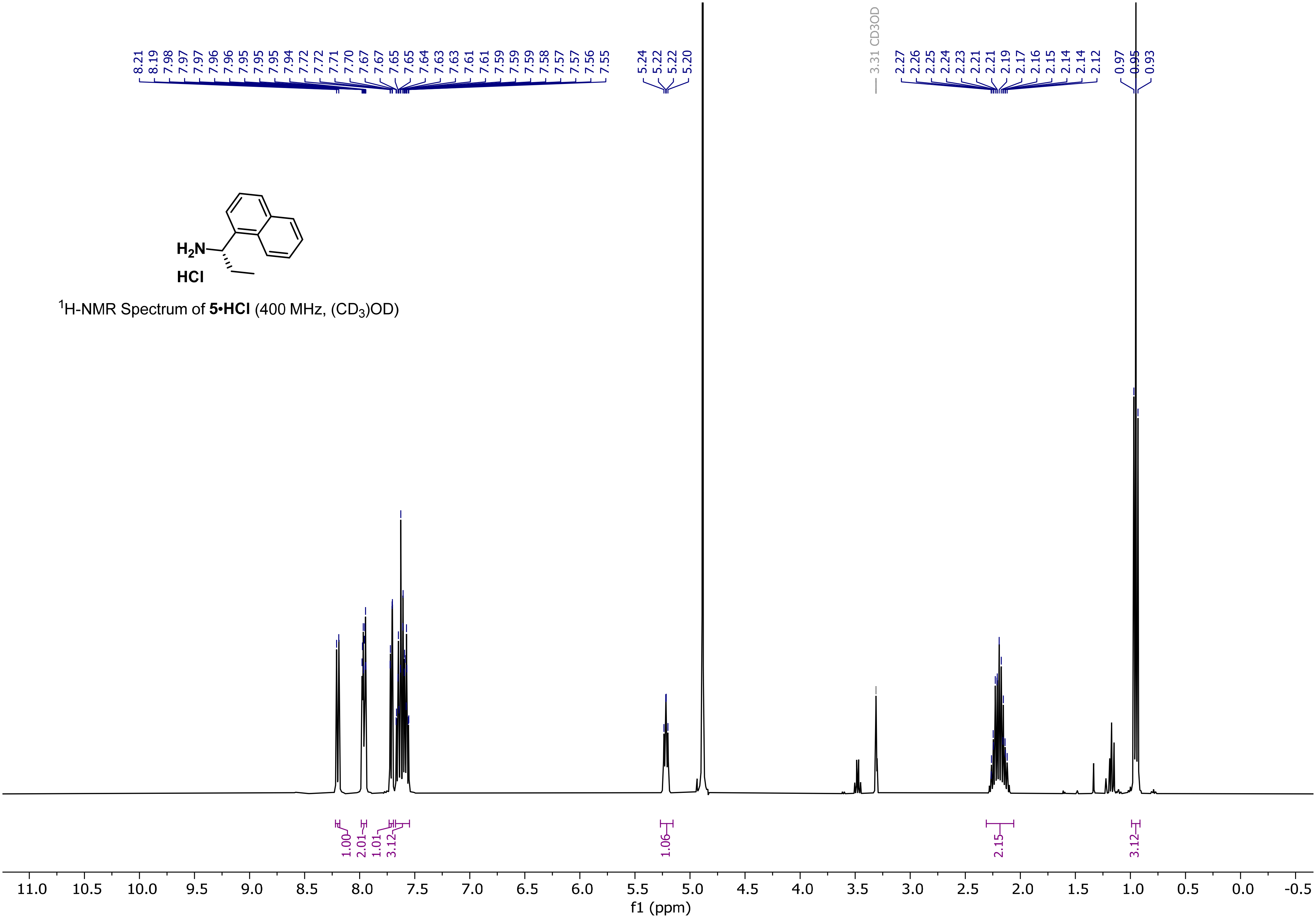

^{1}H-NMR Spectrum of **5•HCl** (400 MHz, $(CD_3)OD$)

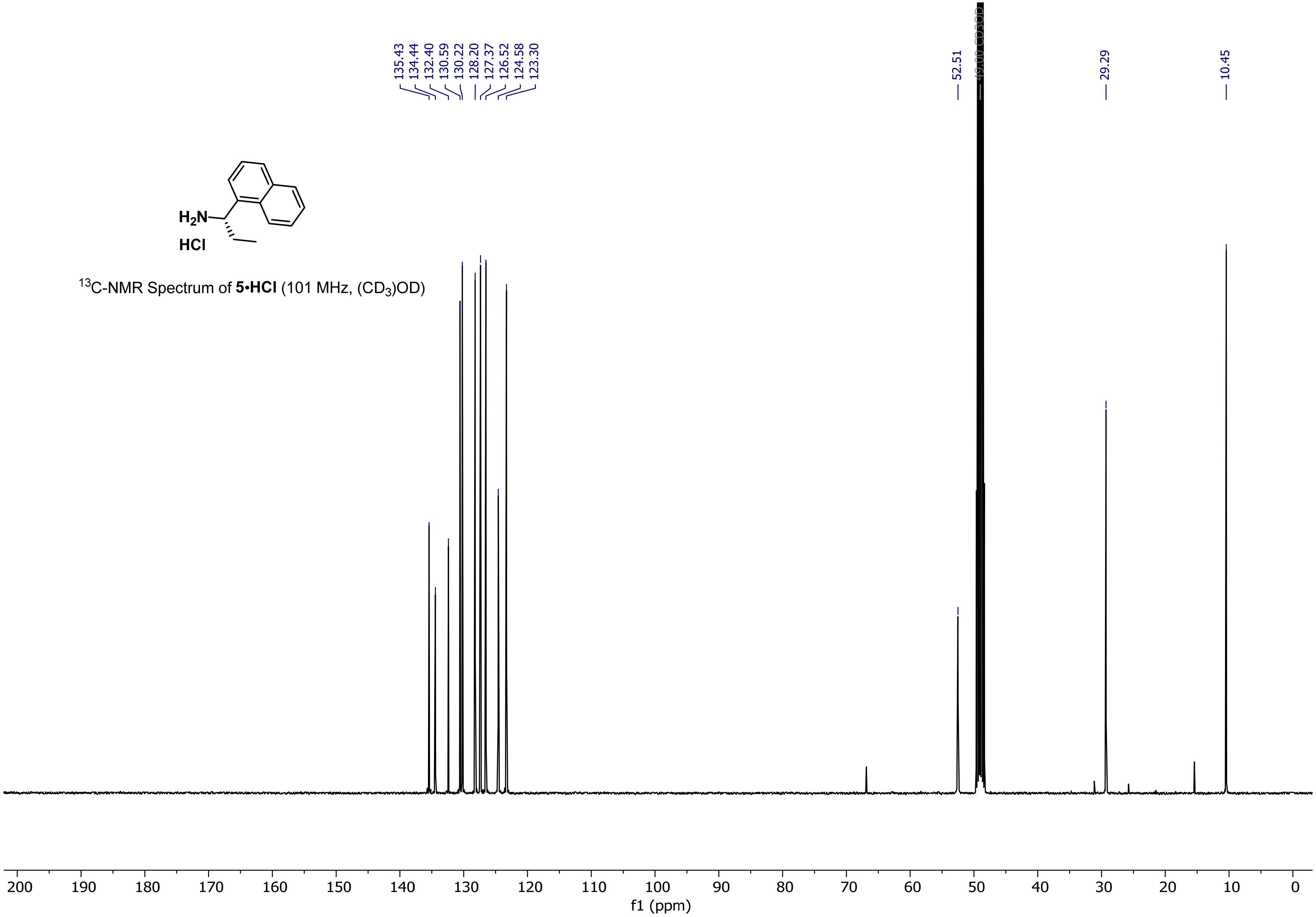

^{13}C-NMR Spectrum of **5•HCl** (101 MHz, $(CD_3)OD$)

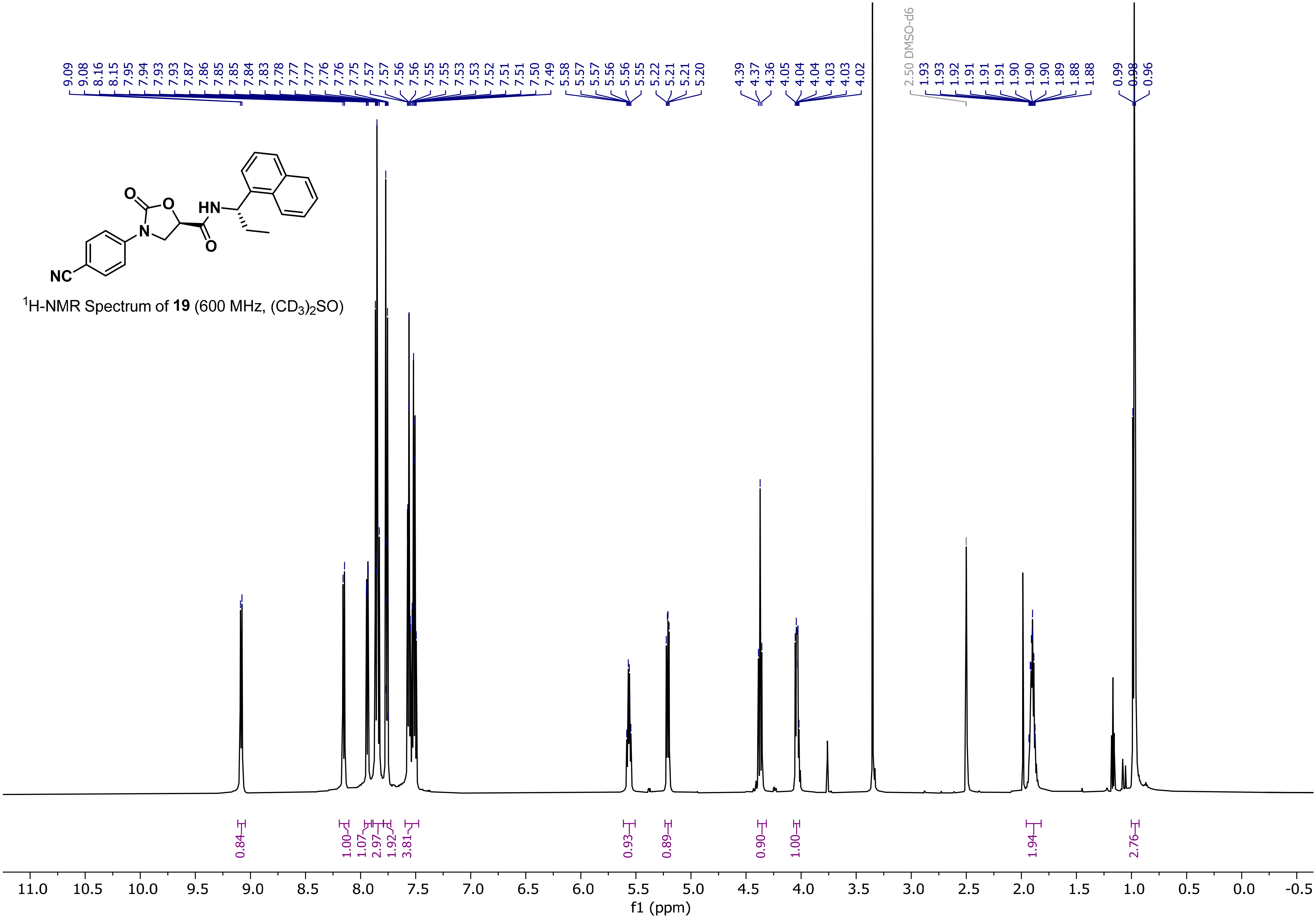

^{1}H-NMR Spectrum of **19** (600 MHz, $(CD_3)_2SO$)

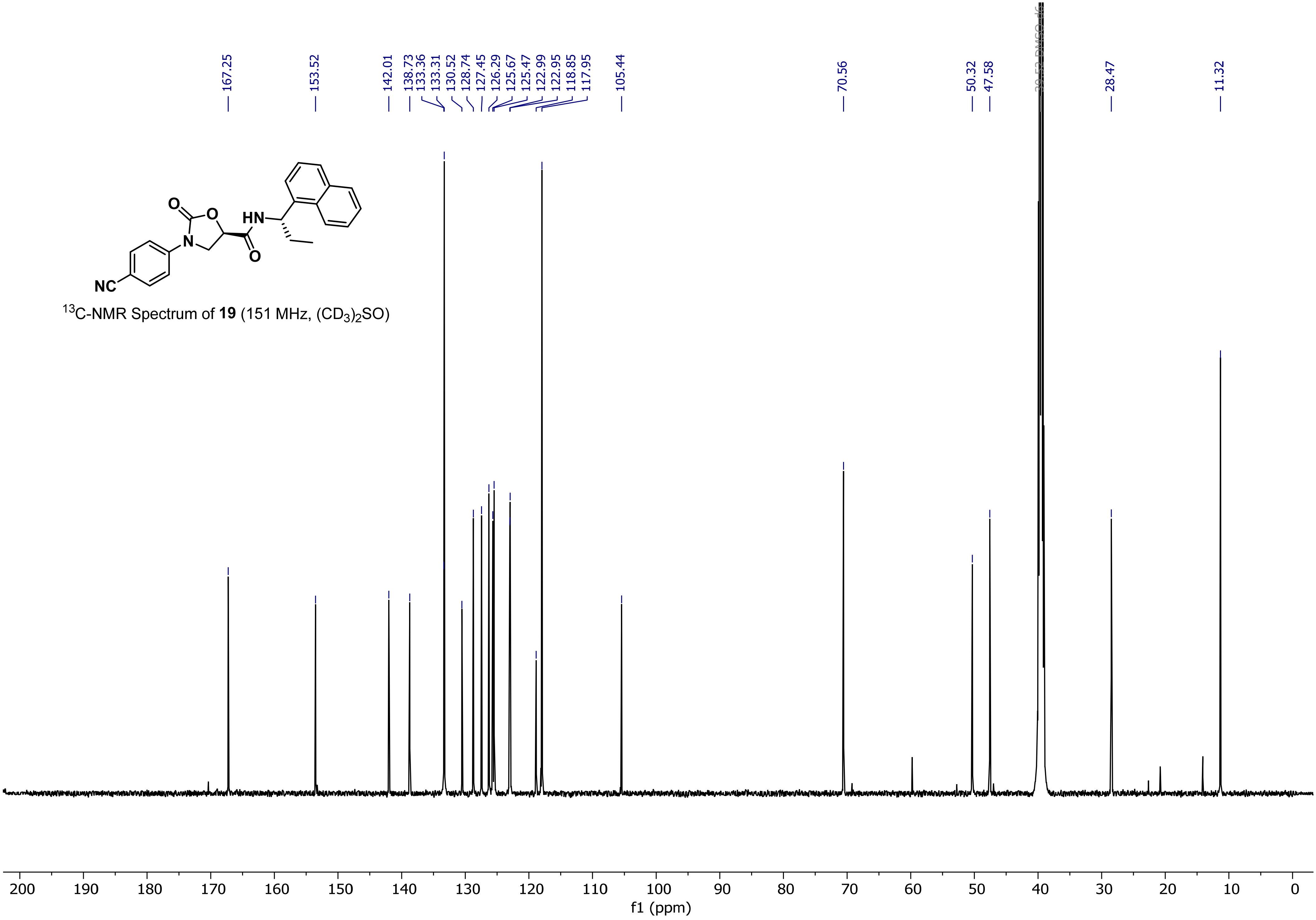

^{13}C-NMR Spectrum of **19** (151 MHz, $(CD_3)_2SO$)

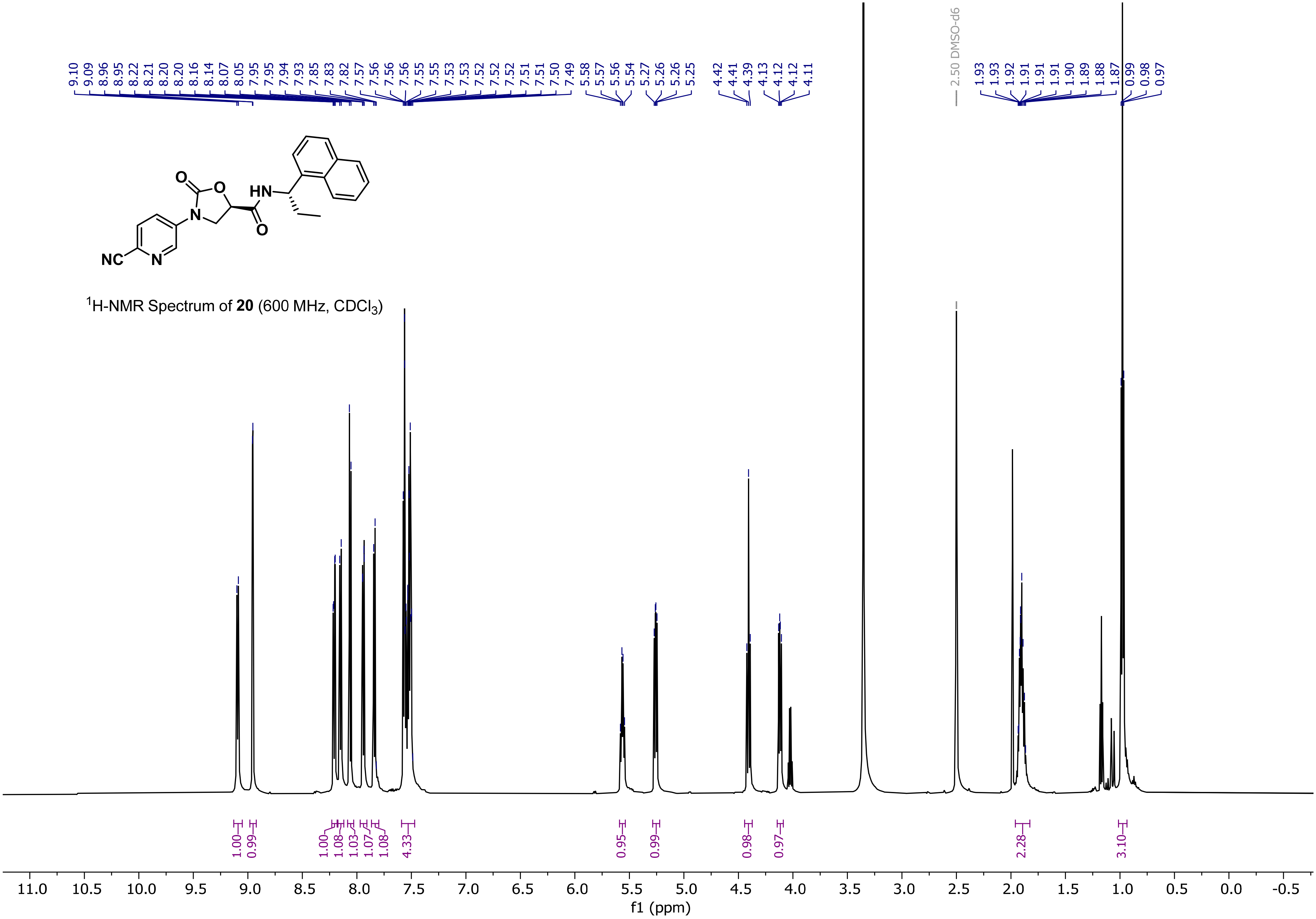

^{1}H-NMR Spectrum of **20** (600 MHz, $CDCl_3$)

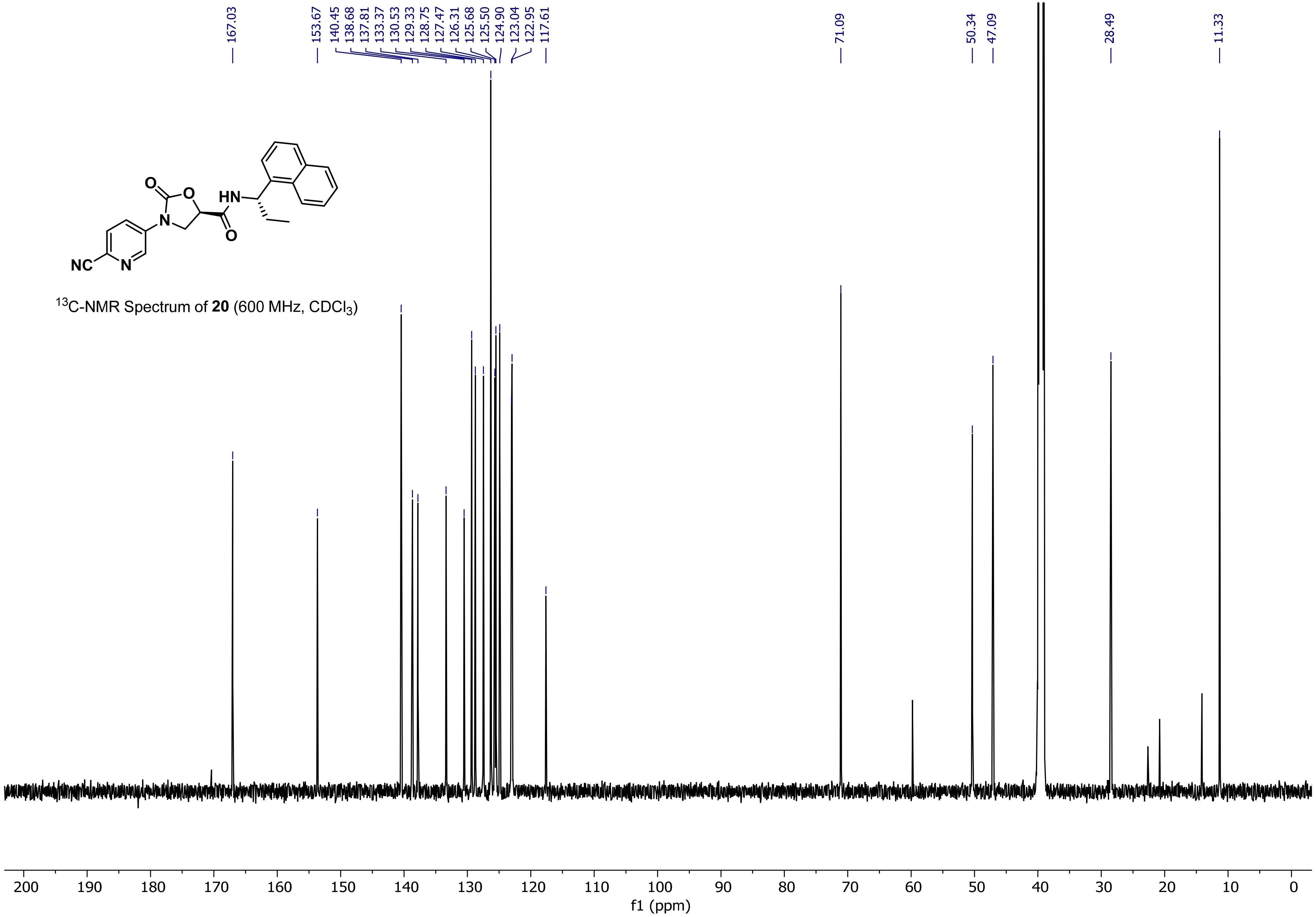

^{13}C-NMR Spectrum of **20** (600 MHz, $CDCl_3$)

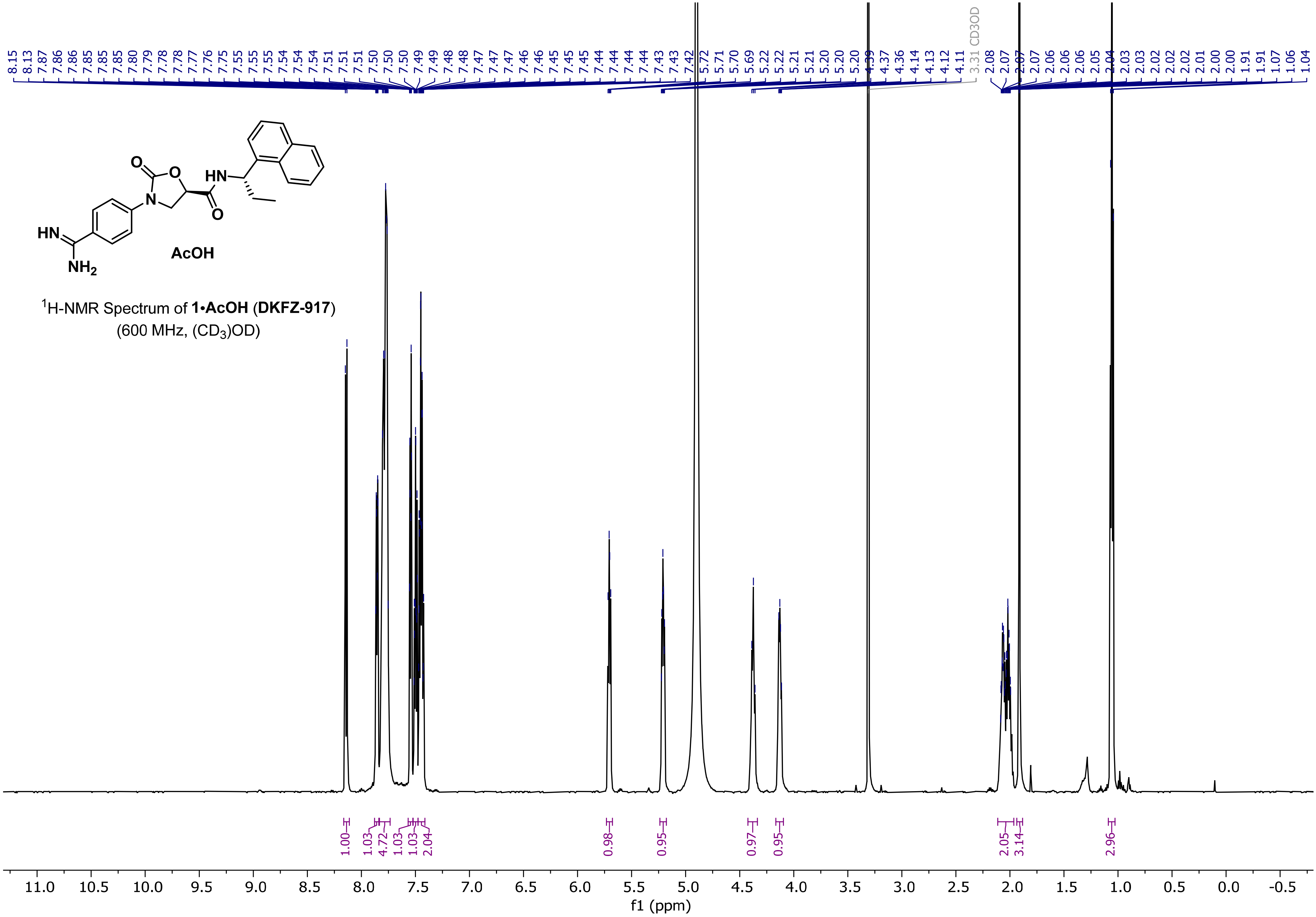

^{1}H-NMR Spectrum of **1•AcOH** (**DKFZ-917**)
(600 MHz, $(CD_3)OD$)

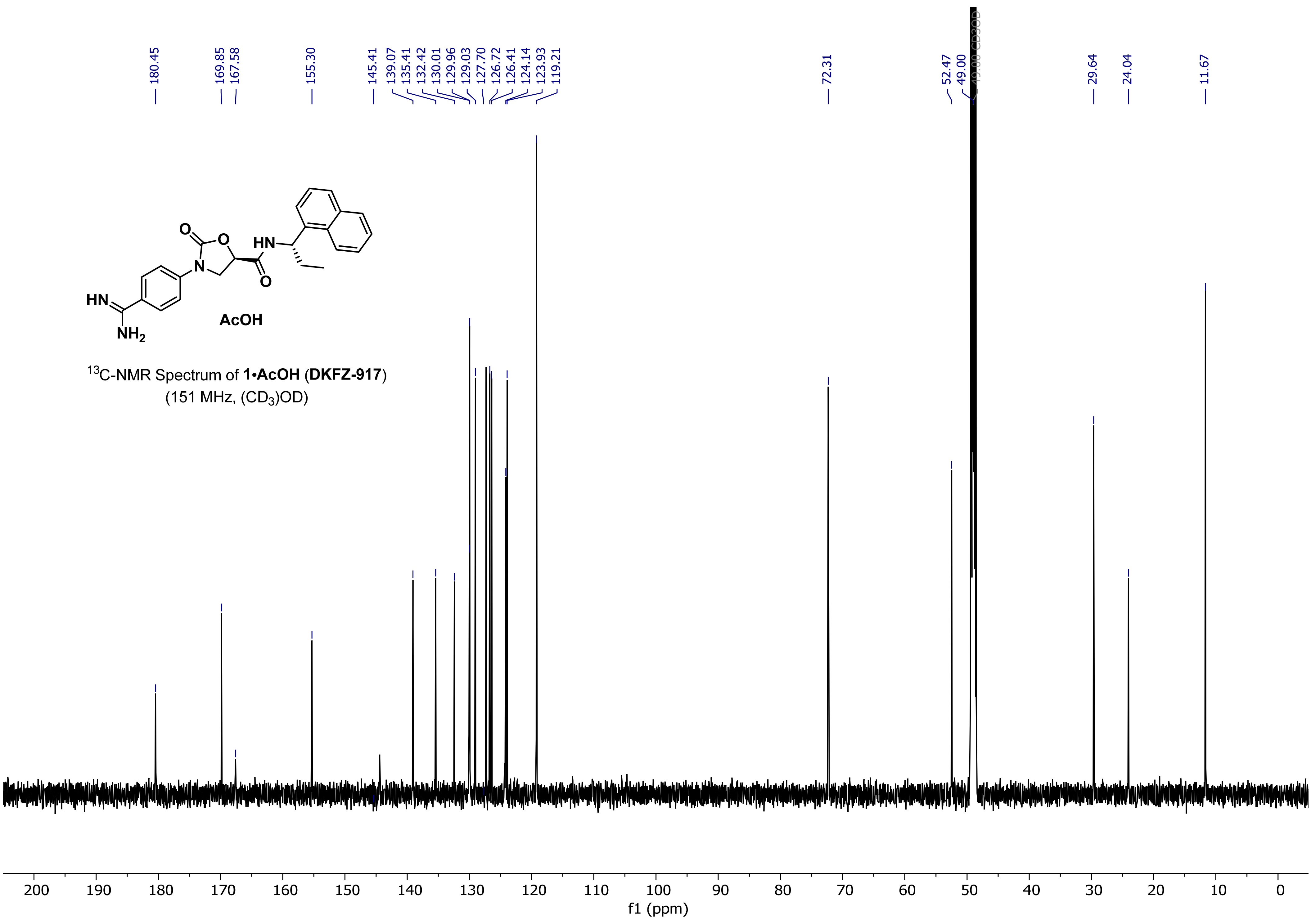

^{13}C-NMR Spectrum of **1•AcOH** (**DKFZ-917**)
(151 MHz, $(CD_3)OD$)

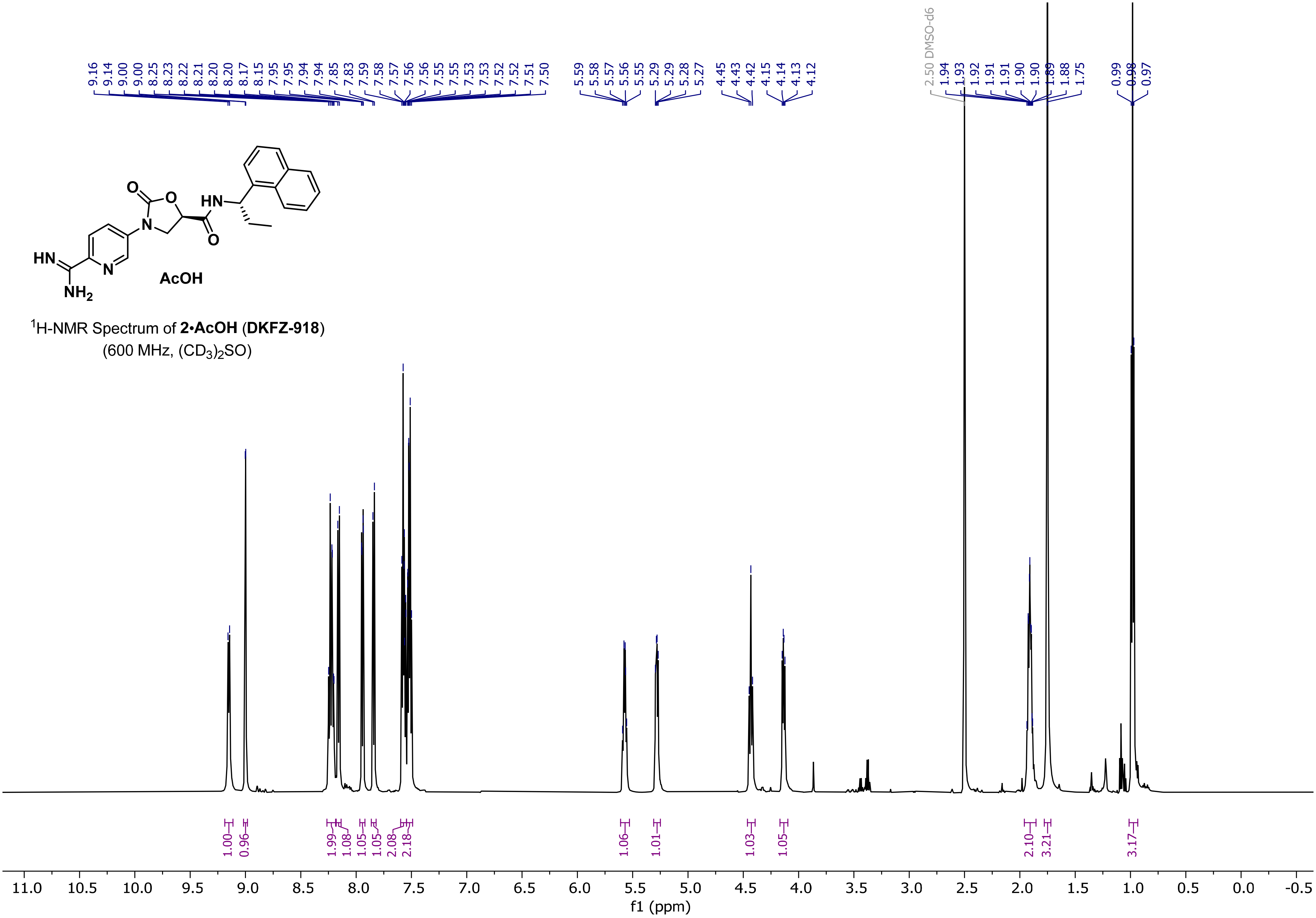

^{1}H-NMR Spectrum of **2•AcOH** (**DKFZ-918**)
(600 MHz, $(CD_3)_2SO$)

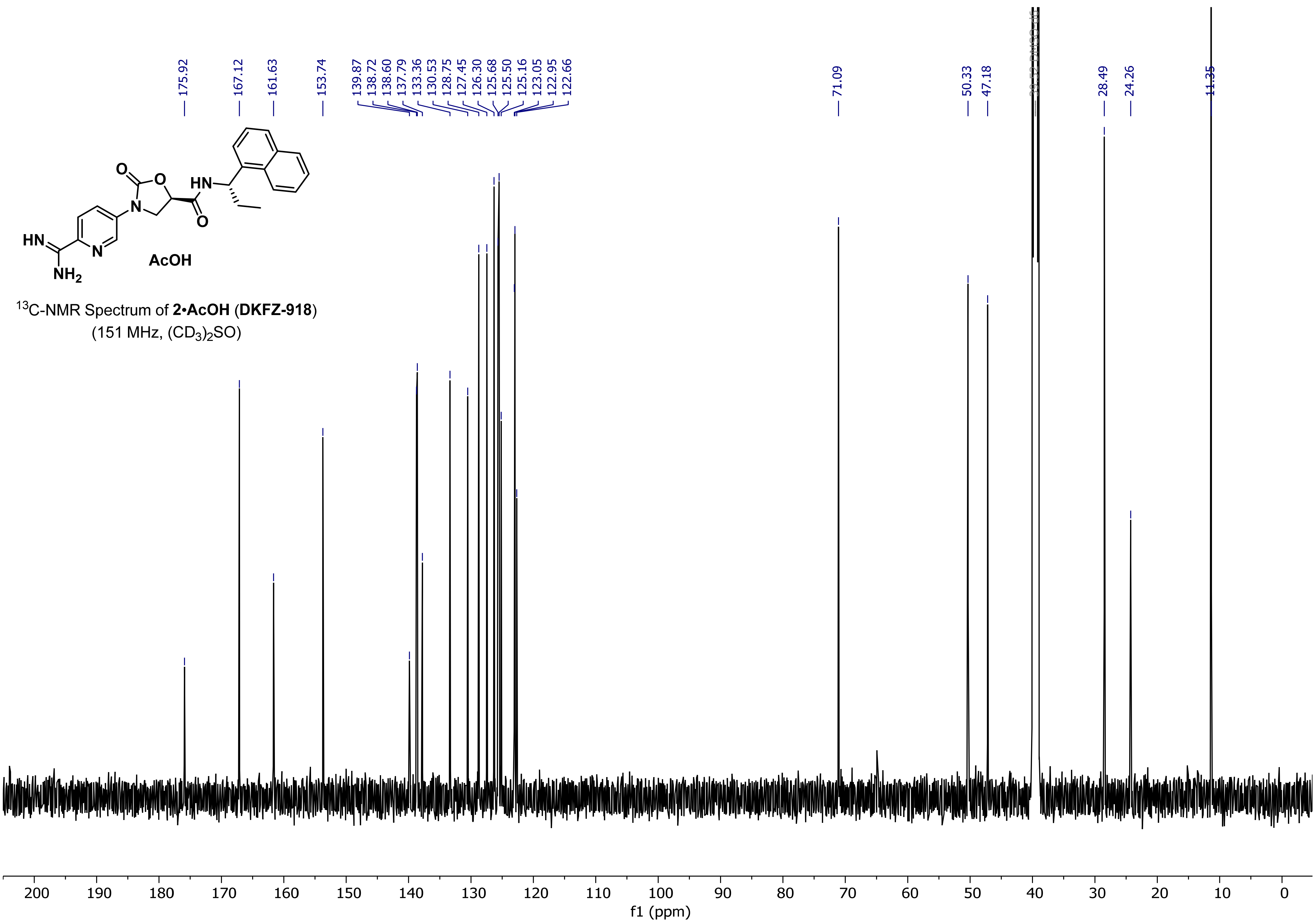

^{13}C-NMR Spectrum of **2•AcOH** (**DKFZ-918**)
(151 MHz, $(CD_3)_2SO$)
